# Supplementary material for: Synthesis and Pro-Apoptotic Effects of Nitrovinylanthracenes and Related Compounds in Chronic Lymphocytic Leukaemia (CLL) and Burkitt’s Lymphoma (BL)
Source: Molecules. 2023 Dec 14;28(24):8095. doi: 10.3390/molecules28248095 (PMC10746112; doi:10.3390/molecules28248095)
Supplement: Supplementary file 1 [file molecules-28-08095-s001.zip › molecules-2710010-supplementary.pdf]

Supplementary information

Synthesis and pro-apoptotic effects of nitrovinylanthracenes and related compounds in chronic lymphocytic leukaemia (CLL) and Burkitt's lymphoma (BL)

Andrew J. Byrne[1], Sandra A. Bright[2], James. P. McKeown[1], Adam Bergin[1], Brendan Twamley[3], Anthony M. McElligott[4], Sara Noorani[1], Shubhangi Kandwal[2], Darren Fayne[2], Niamh M. O'Boyle[1]*, D. Clive Williams[2] and Mary J. Meegan[1]

[1]School of Pharmacy and Pharmaceutical Sciences, Trinity Biomedical Sciences Institute, Trinity College Dublin, 152 - 160 Pearse St, Dublin 2, Ireland
[2]School of Biochemistry and Immunology, Trinity Biomedical Sciences Institute, Trinity College Dublin, 152 - 160 Pearse St, Dublin 2, Ireland.
[3]School of Chemistry, Trinity Biomedical Sciences Institute, Trinity College Dublin, 152 - 160 Pearse St, Dublin 2, Ireland.
[4]Discipline of Haematology, School of Medicine, Trinity Translational Medicine Institute, St. James's Hospital and Trinity College, Dublin 8, Republic of Ireland

Corresponding author: Niamh M. O'Boyle, School of Pharmacy and Pharmaceutical Sciences, Trinity Biomedical Sciences Institute, Trinity College Dublin, 152 - 160 Pearse St, Dublin 2, Ireland; Email address: NIOBOYLE@tcd.ie

Chemistry: Experimental details for preparation of nitrovinylanthracenes and related compounds

Figure S1-S20: ^{1}H NMR spectrum of (*E*)-9-Chloro-10-(2-nitrovinyl)anthracene (**19d**)

Figure S2: ^{13}C NMR spectrum of (*E*)-9-Chloro-10-(2-nitrovinyl)anthracene (**19d**)

Figure S3: DEPT-90 spectrum of (*E*)-9-Chloro-10-(2-nitrovinyl)anthracene (**19d**)

Figure S4: HSQC spectrum of (*E*)-9-Chloro-10-(2-nitrovinyl)anthracene **(19d)**

Figure S5: HMBC spectrum of (*E*)-9-Chloro-10-(2-nitrovinyl)anthracene **(19d)**

Figure S6: ^{1}H NMR spectrum of (*E*)-9-chloro-10-(2-nitrobut-1-en-1-yl)anthracene (**19f**)

Figure S7: ^{13}C NMR spectrum of (*E*)-9-chloro-10-(2-nitrobut-1-en-1-yl)anthracene (**19f**)

Figure S8: DEPT 135 NMR spectrum of (*E*)-9-chloro-10-(2-nitrobut-1-en-1-yl)anthracene (**19f**)

Figure S9: ^{1}H NMR spectrum of (*E*)-9-(3-nitroallyl)anthracene (**20a**)

Figure S10: ^{13}C NMR spectrum of (*E*)-9-(3-nitroallyl)anthracene (**20a**)

Figure S11: DEPT 135 NMR spectrum of (*E*)-9-(3-nitroallyl)anthracene (**20a**)

Figure S12: C-H COSY spectrum of (*E*)-9-(3-nitroallyl)anthracene (**20a**)

Figure S131: ^{1}H NMR spectrum of 4-(anthracen-9-yl)-4,5-dihydro-1*H*-1,2,3-triazole (**31**)

Figure S14: ^{13}C NMR spectrum of 4-(anthracen-9-yl)-4,5-dihydro-1*H*-1,2,3-triazole (**31**)

Figure S15: ^{1}H NMR spectrum of (*E*)-((10-chloroanthracen-9-yl)methylene) hydrazine (**32b**)

Figure S16: ^{13}C NMR spectrum of (E)-((10-chloroanthracen-9-yl)methylene) hydrazine (**32b**)

Figure S17: ^{1}H NMR spectrum of 2-((10-chloroanthracen-9-yl)methylene) malononitrile (**23b**)

Figure S18: ^{13}C NMR spectrum of 2-((10-chloroanthracen-9-yl)methylene)malononitrile (**23b**)

Figure S19: ^{1}H NMR spectrum of anthracen-9-yl(azepan-1-yl)methanone (**33c**)

Figure S20: ^{13}C NMR spectrum of anthracen-9-yl(azepan-1-yl)methanone (**33c**)

Figure S21. Schematic packing diagram of **19f** viewed normal to the a-axis, with hydrogen atoms omitted for clarity

Figure S22. Molecular structure of **30a** with atomic displacement shown at 50% probability

Figure S23. Schematic packing diagram of one disordered moiety of **30a** viewed normal to the b-axis, with hydrogen atoms omitted for clarity

Table S1: Tier-1 profiling screen of selected nitrovinylanthracenes and related compounds

Table S2: ADMET and Lipinski properties for selected nitrovinylanthracenes and related compounds

Tables S3-S6: Preliminary cell viability data for nitrovinylanthracenes and related compounds in MUTU-1 (24 h) and DG-75 Burkitt lymphoma (48 h) cell lines

Table S7: *in vitro* anti-proliferative activity of nitrostyrene compounds **11g-11k** in MUTU-1 and DG-75 cell lines

Table S8: *in vitro* anti-proliferative activity of nitrostyrene compound **11h** in peripheral blood mononuclear cells (PBMCs)

Table S9: Overlay of all nitrostyrene and nitrovinylanthracene panel compounds on maprotiline with their overlay scores and BL (MUTI-1) viability

Table S10: Overlay of selected nitrostyrene and nitrovinylanthracene panel compounds on maprotiline with their overlay scores and CLL (HG-3, PGA-1) viability[a]

References

Experimental chemistry

Uncorrected melting points were measured on a Gallenkamp apparatus. Infra-red (IR) spectra were recorded on a Perkin Elmer FT-IR Paragon 1000 spectrometer. ^{1}H, ^{13}C and ^{19}F nuclear magnetic resonance spectra (NMR) were recorded at 27 ^{o}C on a Bruker DPX 400 spectrometer (400.13 MHz, ^{1}H; 100.61 MHz, ^{13}C; 376.47 MHz, ^{19}F) in either $CDCl_3$ (internal standard tetramethylsilane (TMS)) or CD_3OD or DMSO-d_6. For $CDCl_3$, ^{1}H-NMR spectra were assigned relative to the TMS peak at 0.00 ppm and ^{13}C-NMR spectra were assigned relative to the middle $CDCl_3$ peak at 77.0 ppm. For CD_3OD, ^{1}H and ^{13}C-NMR spectra were assigned relative to the center peaks of the CD_3OD multiplets at 3.30 ppm and 49.00 ppm respectively. Coupling constants are reported in Hertz. For ^{1}H-NMR assignments, chemical shifts are reported: shift value (number of protons, description of absorption, coupling constant(s) where applicable). Electrospray ionisation mass spectrometry (ESI-MS) was performed in the positive ion mode on a liquid chromatography time-of-flight mass spectrometer (Micromass LCT, Waters Ltd., Manchester, UK). The samples were introduced to the ion source by an LC system (Waters Alliance 2795, Waters Corporation, USA) in acetonitrile:water (60:40 %v/v) at 200 µL/min. The capillary voltage of the mass spectrometer was at 3kV. The sample cone (de-clustering) voltage was set at 40V. For exact mass determination, the instrument was externally calibrated for the mass range m/z 100 to m/z 1000. A lock (reference) mass (m/z 556.2771) was used. Mass measurement accuracies of <±5 ppm were obtained. R_f values are quoted for thin layer chromatography on silica gel Merck F-254 plates, unless otherwise stated. Flash column chromatography was carried out on Merck Kieselgel 60 (particle size 0.040-0.063mm). Microwave experiments were carried out using the Discover CEM microwave synthesiser on standard power setting (300 watts) unless otherwise stated.

General procedure for synthesis of nitrostyrenes 11a-l [1].

To a solution of the appropriate benzaldehyde (3.6 mmol) in a 5 mL microwave tube, nitroethane or nitropropane (7.2 mmol) was added. Glacial acetic acid (4 mL) was transferred into the mixture, followed by cyclohexylamine (3.6 mmol). The tube was sealed and the reaction was heated under microwave irradiation. The mixture was heated at 120 ^{o}C for 30 min with standard power. Once complete, the reaction was allowed to stand and cool. Water (10 mL) was added and the reaction mixture was cooled over ice. The precipitated product was collected by filtration. Water (20 mL) was added to the

filtrate and any remaining nitrostyrene product was extracted using 3x10 mL washes of DCM and $NaHCO_3$. The separated organic phase was dried over anhydrous Na_2SO_4, and the remaining solvent was removed under reduced pressure. The filtered and extracted nitroethane products were combined and purified by flash chromatography and recrystallized from methanol or ethanol.

(*E*)-1-Fluoro-2-(2-nitroprop-1-en-1-yl)benzene (11a)

(*E*)-1-Fluoro-2-(2-nitroprop-1-en-1-yl)benzene was prepared from 2-fluorobenzaldehyde (3.6 mmol) following the general procedure above [1, 34] and purified by flash chromatography (eluent 3:1 DCM/hexane). The product was recrystallized from methanol as yellow crystals, 0.412 g, yield 65.1%, Mp 47-49 ^oC [35] IR v max: 3085.64, 2938.17 (C-H), 1649.62 (C=C), 1517.06, 1312.24 (NO_2), 1090.34 (C-N) cm^{-1}. ^1H NMR (400 MHz, $CDCl_3$) δ 2.38 (s, 3H, -CH_3), 7.20 – 7.36 (br, s, 3H, 3 x ArH), 7.33 – 7.45 (br. s, 1H), 8.11 (s,1H, H1). ^{13}C NMR ($CDCl_3$): 161.76, 143.97, 131.83, 130.26, 126.56, 124.33, 116.22, 116.01, 14.19 ppm.

(*E*)-1-Fluoro-3-(2-nitroprop-1-en-1-yl)benzene (11b)

(*E*)-1-Fluoro-3-(2-nitroprop-1-en-1-yl)benzene was prepared from 3-fluorobenzaldehyde (3.6 mmol) following the general procedure above, and purified by flash chromatography (eluent 3:1 DCM/hexane). The product was obtained as a yellow oil, 0.236 g, yield 37.2%. [35] IR v max: 3070.01, 2992.55 (C-H), 1660.08 (C=C), 1516.08, 1323.70 (NO_2), 1150.66 (C-N) cm^{-1}. ^1H NMR (400 MHz, $CDCl_3$) δ 2.44 (s, 3H, -CH_3), 7.11 (m, 1H), 7.20 (m, 3H, 3 x ArH), 8.01 (s,1H, H1). ^{13}C NMR ($CDCl_3$): 163.92, 148.63, 134.40, 132.06, 130.48, 125.74, 116.72, 116.35, 13.97 ppm.

(*E*)-1-Fluoro-4-(2-nitroprop-1-en-1-yl)benzene (11c)

(*E*)-1-Fluoro-4-(2-nitroprop-1-en-1-yl)benzene was prepared from 4-fluorobenzaldehyde (3.6 mmol, 0.459 g) according to general procedure above and recrystallised from ethanol as yellow crystals, 324 mg (50%), Mp. 61-62 ^oC [1] IRν_{max} (KBr): 3079 (CH), 1653 (C=C), 1518, 1354 (NO_2) cm^{-1}. ^1H NMR (400 MHz, $CDCl_3$) δ 2.46 (s, 3 H, CH_3), 7.12-7.22 (m, 2 H, 2 x ArH), 7.40-7.50 (m, 2 H, 2 x ArH), 8.07 (s, 1 H, =CH). ^{13}C NMR (101 MHz, $CDCl_3$) 13.5 (C3), 115.6, 115.8, 128.0, 131.6, 132.03 (C1), 147.0 (C2), 162.9 (CF) ppm. HRMS (ESI) calculated for $C_9H_7FNO_2$ [M^+–H] 180.0455: found 180.0459.

(*E*)-1-Fluoro-2-(2-nitrobut-1-en-1-yl)benzene 11d

(E)-1-Fluoro-2-(2-nitrobut-1-en-1-yl)benzene was prepared from 2-fluorobenzaldehyde (3.6 mmol) following the general procedure above [1, 34], and purified by flash chromatography (eluent 3:1 DCM/hexane). The product was recrystallized from methanol as yellow crystals 0.224 g, yield 32.8%, Mp 48-50 ^{o}C. IR v max: 2979.57, 2881.60 (C-H), 1611.48 (C=C), 1519.40, 1324.10 (NO_2), 1161.28 (C-N) cm^{-1}. ^{1}H NMR (400 MHz, $CDCl_3$) δ 1.24 (t, J = 7.26 Hz, 3H, -CH_3), 2.72 – 2.84 (q, J = 7.33 Hz, 2H), 7.12 – 7.18 (m, 1H, 1 x ArH), 7.20 – 7.26 (m, 2H, 2 x ArH), 7.29-7.48 (m, 1H, 1 x ArH), 8.04 (s,1H, H1). ^{13}C NMR ($CDCl_3$): 12.59, 21.29, 116.53, 124.79, 126.24, 130.06, 132.16, 155.09, 159.68 ppm

(E)-1-Fluoro-3-(2-nitrobut-1-en-1-yl)benzene (11e)

(E)-1-Fluoro-3-(2-nitrobut-1-en-1-yl)benzene was prepared from 3-fluorobenzaldehyde (3.6 mmol) following the general procedure above [1], and purified by flash chromatography (eluent 3:1 DCM/hexane). The product was recrystallized from methanol as yellow crystals 0.301 g, yield 44.1%, Mp 39-40 ^{o}C. IR v max: 2973.34, 2878.13 (C-H), 1653.28 (C=C), 1514.08, 1326.41 (NO_2), 1153.91 (C-N) cm^{-1}. ^{1}H NMR (400 MHz, $CDCl_3$) δ 1.26 (t, 3H, -CH_3), 2.83 (q, 2H), 7.12 – 7.42 (m, 3H, 3 x ArH), 7.94 (s,1H, H1). ^{13}C NMR ($CDCl_3$): 165.81, 152.95, 134.36, 131.57, 130.54, 125.40, 116.75, 116.03, 20.68, 12.43 ppm.

(E)-1-Fluoro-4-(2-nitrobut-1-en-1-yl)benzene (11f)

(E)-1-Fluoro-4-(2-nitrobut-1-en-1-yl)benzene was prepared from 4-fluorobenzaldehyde (3.6 mmol, 0.459 g) according to general procedure above and recrystallised from ethanol as yellow crystals 280 mg (40%), Mp. 79-80 ^{o}C [1]. IRν_{max} (KBr): 2967 (CH), 1653 (C=C), 1508, 1323 (NO_2) cm^{-1}. ^{1}H NMR (400 MHz, $CDCl_3$) δ 1.29 (t, 3 H, J = 7.5 Hz, -CH_3), 2.86 (q, 2 H, J = 7 Hz, -CH_2), 7.17 (m, 2 H, 2 x ArH), 7.43 (m, 2 H, 2 x ArH), 8.00 (1H, s, =CH). ^{13}C NMR (101 MHz, $CDCl_3$) 12.0 (C4), 20.2 (C3), 115.7, 115.9, 127.9, 131.4, 131.6 (C1), 152.6 (C2), 163.1 (CF) ppm. HRMS (ESI) calculated for $C_{10}H_{10}FNO_2$ [M^+] 195.0690: found 195.0686.

(E)-1-Chloro-2-(2-nitroprop-1-en-1-yl)benzene (11g)

(E)-1-Chloro-2-(2-nitroprop-1-en-1-yl)benzene was prepared from 2-chlorobenzaldehyde (1 mmol, 0.14 g) according to general procedure above. The product was purified by column chromatography (eluent: 1:1 dichloromethane: hexane)., to afford a yellow oil, [1] 110 mg (56%). IR$_{Vmax}$ (ATR): 3068, 2934 (C-H),

1665 (C=C), 1518, 1324 (NO_2), 1055 (C-N) cm^{-1}. ^{1}H NMR (400 MHz, $CDCl_3$) δ 2.30 (s, 3 H, CH_3), 7.27 - 7.39 (m, 3 H, 3 x ArH), 7.41 - 7.47 (m, 1 H, H6), 8.06 - 8.20 (m, 1 H, H1'). ^{13}C NMR (101 MHz, $CDCl_3$) 13.7 (CH_3), 124.3 (CH), 126.8 (CH), 129.8 (CH), 130.1 (CH), 130.4 (CH), 130.8, 131.0, 134.6, 149.1 (C2') ppm. HRMS (APCI) calculated for $C_9H_9NO_2Cl$ [M^++H] 198.0322: found 198.0321.

(*E*)-1-Chloro-3-(2-nitroprop-1-en-1-yl)benzene (11h)

(*E*)-1-Chloro-3-(2-nitroprop-1-en-1-yl)benzene was prepared from 3-chlorobenzaldehyde (1 mmol, 0.14 g) according to general procedure above. The product was purified by column chromatography (eluent: 1:1 dichloromethane: hexane) to afford a yellow oil [1], 120 mg (61%). IR$_{Vmax}$ (ATR): 3067, 2926 (Ar C-H), 1660, 1594 (Ar C=C), 1519, 1323 (NO_2), 1168 (C-N) cm^{-1}. ^{1}H NMR (400 MHz, $CDCl_3$) δ 2.45 (s, 3 H, CH_3), 7.30 - 7.35 (m, 1 H, 1 x ArH), 7.41 (d, *J* = 3.73 Hz, 3 H, 3 x ArH), 8.01 (s, 1 H, H1'). ^{13}C NMR (101 MHz, $CDCl_3$) 13.9 (CH_3), 124.3 (CH), 129.5 (CH), 129.8 (CH), 130.1 (CH), 131.8 (C1'), 134.1, 134.8, 148.7 (C2') ppm. HRMS (APCI) calculated for $C_9H_8NO_2Cl$ [M^+] 197.0244: found 197.0242.

(*E*)-1-Chloro-4-(2-nitroprop-1-en-1-yl)benzene (11i)

(*E*)-1-Chloro-4-(2-nitroprop-1-en-1-yl)benzene was prepared from 4-chlorobenzaldehyde (3.6 mmol, 0.505 g) according to general procedure above. The product was purified by flash column chromatography over silica gel (eluent: 3:1 dichloromethane/hexane) and recrystallised from ethanol as yellow crystals, 486 mg (64%)**,** Mp. 82-83 °C [1]. IRν_{max} (KBr): 3053 (CH), 1641 (C=C), 1526, 1353 (NO_2), 831 (C-Cl) cm^{-1}. ^{1}H NMR (400 MHz, $CDCl_3$) δ 2.44 (s, 3 H, CH_3), 7.38 (d, 2 H, *J* = 9 Hz, 2 x ArH), 7.43 (d, 2 H, *J* = 9 Hz, 2 x ArH), 8.03 (s, 1 H, H1). ^{13}C NMR (101 MHz, $CDCl_3$) 13.6 (C3), 128.8, 130.4, 131.8, 131.8, 135.6 (C1), 147.6 (C2) ppm. HRMS (ESI) calculated for $C_9H_9ClNO_2$ [M^++H] 198.0322: found 198.0314.

(*E*)-1-Chloro-2-(2-nitrobut-1-en-1-yl)benzene (11j)

(*E*)-1-Chloro-2-(2-nitrobut-1-en-1-yl)benzene was prepared from 2-chlorobenzaldehyde (1 mmol, 0.14 g) according to general procedure above. The product was purified by flash column chromatography over silica gel (eluent: dichloromethane : hexane 3/1) to afford a yellow oil [1]. 199 mg (94%). IR$_{Vmax}$ (ATR): 3066, 2939 (C-H), 1659, 1591 (C=C), 1521, 1330 (NO_2), 1128 (C-N) cm^{-1}. ^{1}H NMR (400 MHz, $CDCl_3$) δ 1.12 (t, *J* = 7.32 Hz, 3 H, CH_3), 2.60 - 2.67 (m, 2 H, CH_2), 7.20 - 7.32 (m, 3 H, 3 x ArH), 7.32 - 7.40 (m, 1 H, H6), 7.98 (s, 1 H, H1'). ^{13}C NMR (101

MHz, $CDCl_3$) 12.1 (CH_3), 20.3 (CH_2), 126.8 (CH), 129.4 (CH), 129.5 (CH), 129.7 (CH), 130.6 (C1'), 130.8, 134.2, 154.3 (C1) ppm. HRMS (APCI) calculated for $C_{10}H_{11}ClNO_2$ [M^++H] 212.0478: found 212.0476.

(*E*)-1-Chloro-3-(2-nitrobut-1-en-1-yl)benzene (11k)

(*E*)-1-Chloro-3-(2-nitrobut-1-en-1-yl)benzene was prepared from 3-chlorobenzaldehyde (1 mmol, 0.14 g) according to general procedure above. The product was purified by flash column chromatography over silica gel (eluent: dichloromethane : hexane 3:1), to afford a yellow oil 49 mg [1]. IR$_{Vmax}$ (ATR): 3066, 2939.63 (C-H), 1657, 1621 (C=C), 1520, 1330 (NO_2), 1168 (C-N) cm^{-1}. ^{1}H NMR (400 MHz, $CDCl_3$) δ 1.21 (t, *J* = 7.63 Hz, 3 H, CH_3), 2.75 - 2.84 (m, 2 H, CH_2), 7.21 - 7.28 (m, 1 H, H2), 7.28 - 7.41 (m, 3 H, 3 x ArH), 7.85 (s, 1 H, H1'). ^{13}C NMR (101 MHz, $CDCl_3$) 12.2 (CH_3), 20.5 (CH_2), 127.4 (CH), 129.1 (CH), 129.7 (CH), 130.1 (CH), 131.2 (C1'), 134.0, 134.7, 154.1 (C2') ppm. HRMS (APCI) calculated for $C_{10}H_{10}NO_2Cl$ [M^+] 211.0395: found 211.0399.

(*E*)-1-Chloro-4-(2-nitrobut-1-en-1-yl)benzene (11l)

(*E*)-1-Chloro-4-(2-nitrobut-1-en-1-yl)benzene was prepared from 4-chlorobenzaldehyde (3.6 mmol, 0.50 g) using according to general procedure above. The product was purified by flash column chromatography over silica gel (eluent: 3:1 dichloromethane / hexane) and recrystallised from ethanol as yellow crystals 151 mg (20%), Mp. 75-77 ^{o}C [1]. IR$_{vmax}$ (KBr): 2976 (CH), 1651 (C=C), 1520, 1382 (NO_2), 836 (C-Cl) cm^{-1}. ^{1}H NMR (400 MHz, $CDCl_3$) δ 1.29 (t, 3 H, *J* = 7.5 Hz, -CH_3), 2.86 (q, 2 H, *J* = 7.5 Hz, -CH_2), 7.37 (d, 2 H, *J* = 8.5 Hz, 2 x ArH), 7.44 (d, 2 H, *J* = 8.5 Hz, 2 x ArH), 7.99 (s, 1 H, H1). ^{13}C NMR (101 MHz, $CDCl_3$) 12.0 (C4), 20.3 (C3), 128.9, 130.3, 130.4, 131.4 (C1), 153.2 (C2) ppm. HRMS (APCI) calculated for $C_{10}H_{10}NO_2Cl$ [M^++H] 211.0395: found 211.0389.

9-Ethylanthracene (13a)

To a hot solution of anthrone (2 g, 10.3 mmol) in anhydrous toluene (20 mL) was added portion-wise a solution of 3.0 M ethyl magnesium bromide (3.8 mL) in THF (11.33 mmol). The reaction was heated to reflux under nitrogen for 2 h until the mixture turned green. The mixture was allowed to cool to room temperature and poured over a slurry of 20 % HCl and crushed ice (50 mL). Once the ice had melted; the aqueous layer was extracted into toluene and the solvent was removed *in vacuo*. The crude product was washed with hot water and recrystallized from ethanol to afford a yellow solid 1.71 g

(80%), Mp. 55-58 °C (lit. Mp. 59 °C [4]). IR$_{Vmax}$ (ATR): 3025, 2977 (C-H), 1621 (C=C), 1494, 1443 (C=C) cm^{-1}. ^{1}H NMR (400 MHz, $CDCl_3$) δ 1.47 - 1.52 (m, 3 H, CH_3), 3.68 (q, *J* = 7.32 Hz, 2 H, CH_2), 7.44 - 7.61 (m, 4 H, 4 x ArH), 8.04 (d, *J* = 8.55 Hz, 2 H, 2 x ArH), 8.26 - 8.41 (m, 3 H, 2 x ArH, H10). ^{13}C NMR (101 MHz, $CDCl_3$) 15.5 (CH_3), 21.1 (CH_2), 123.5 (CH), 124.3 (CH), 124.8 (CH), 125.3 (CH), 125.4 (CH), 125.5 (CH), 126.2 (CH), 128.1 (CH), 129.2, 129.2 (CH), 131.7, 136.6 ppm. HRMS (APCI) calculated for $C_{16}H_{15}$ [M^++H] 207.1174: found 207.1168.

9-Isopropylanthracene (13b)

To a hot solution of anthrone (2 g, 10.3 mmol) in anhydrous toluene (20 mL) was added portion-wise isopropyl magnesium bromide (8.3 mL of 3.0 M solution, 11.33 mmol) in diethyl ether. The reaction was heated to reflux under nitrogen for 2 h until the mixture turned green. The mixture was allowed to cool to room temperature and poured over a slurry of 20 % HCl and crushed ice. Once the ice had melted the aqueous layer was extracted into toluene and the solvent was removed *in vacuo*. The crude product was washed with hot water and recrystallized from ethanol to afford a yellow solid, 1.72 g (76%), Mp. 61-65 °C (lit. Mp. 76 °C [5]). IR$_{Vmax}$ (ATR): 3069, 2988 (C-H), 1591 (C=C), 1485, 1442 (C=C) cm^{-1}. ^{1}H NMR (400 MHz, $CDCl_3$) δ 1.78 (d, *J* = 7.05 Hz, 6 H, 2 x CH_3), 4.60 (s, 1 H, CH), 7.41 - 7.53 (m, 5 H, 5 x ArH), 8.02 (dd, *J* = 8.50, 1.45 Hz, 3 H, 3 x ArH), 8.35 (s, 1 H, H10). ^{13}C NMR (101 MHz, $CDCl_3$) 22.9 (CH_3), 28.3 (CH), 110.0 (CH), 124.5 (CH), 125.3 (CH), 126.2 (CH), 126.3 (CH), 127.2 (CH), 128.1 (CH), 129.5 (CH), 131.6, 134.1 (CH) ppm. HRMS (APCI) calculated for $C_{17}H_{16}$ [M^+] 220.1252: found 220.1248.

9-Phenylanthracene (13c)

To a solution of anthrone (4 g, 20.6 mmol) in anhydrous toluene (20 mL) under nitrogen, was added portion-wise, a solution of phenylmagnesium bromide (3.0 M, 24.72 mmol, 8.3 mL) in diethyl ether. The reaction mixture was refluxed under nitrogen for 3 h until the reaction mix turned green. The mixture was allowed to cool to room temperature and poured over a slurry of 20 % HCl and crushed ice (50 mL). The organic layer was separated and solvent was removed *in vacuo*. Product was purified by column chromatography (eluent dichloromethane: hexane (1:4)) to afford a yellow solid 4.41 g (84%), Mp. 150-153 °C (lit. Mp. 153-154 °C [6]). IR$_{Vmax}$ (ATR): 3029, 2998 (C-H), 1667 (C=C), 1511, 1486 (C=C) cm^{-1}. ^{1}H NMR (400 MHz, $CDCl_3$) δ 7.40 - 7.47 (m, 2 H, 2 x ArH), 7.50 - 7.57 (m, 4 H, 4 x ArH), 7.60 - 7.70 (m, 3 H, 3 x ArH), 7.79 (d, *J* = 8.71 Hz, 2 H, 2 x ArH), 8.12 (d, *J* = 8.29 Hz, 2 H, 2 x ArH), 8.57 (s, 1 H, H10). ^{13}C

NMR (101 MHz, $CDCl_3$) 125.0 (CH), 125.3 (CH), 126.5 (CH), 126.8 (CH), 127.4 (CH), 128.3 (CH), 128.3 (CH), 130.2, 131.2 (CH), 131.3, 136.9, 138.7 (C9) ppm. HRMS (APCI) calculated for $C_{20}H_{15}$ [M^++H] 255.1168: found 255.1158.

9-Methoxyanthracene (13d)

To a stirred solution of anthrone (1.94 g, 10 mmol) in methanol (30 mL) and benzene (30 mL), was added trimethyl orthoformate (1.06 g) and concentrated sulfuric acid (10 drops). The reaction mixture was heated to reflux for 3 days. The reaction was cooled and poured onto saturated sodium hydrogen carbonate solution (100 mL) and extracted with ether. The organic extracts were washed with water, brine and dried over Na_2SO_4. Solvent was removed *in vacuo* and the solid was recrystallized from ethanol as a pale yellow solid 1.59 g (76%), Mp. 90-94 ^oC (lit. Mp. 94 ^oC [2]). IR$_{Vmax}$ (ATR): 3051, 2969 (C-H), 1620 (C=C), 1571, 1462 (C=C) cm^{-1}. ^1H NMR (400 MHz, $CDCl_3$) δ 4.18 (s, 3 H, OCH_3), 7.50 (dqd, J = 8.05, 6.27, 6.27, 6.27, 1.83 Hz, 4 H, 4 x ArH), 7.97 - 8.06 (m, 2 H, 2 x ArH), 8.25 (s, 1 H, H10), 8.29 - 8.38 (m, 2 H, 2 x ArH). ^{13}C NMR (101 MHz, $CDCl_3$) 63.1 (CH_3), 122.2 (CH), 122.2 (CH), 124.4, 125.1 (CH), 125.4 (CH), 128.4 (CH), 132.4, 152.2 (C9) ppm. HRMS (APCI) calculated for $C_{15}H_{13}O$ [M^++H] 209.0966: found 209.0958.

9-Methylanthracene (13e)

To a mixture of 9-anthraldehyde (2 g, 9.7 mmol), hydrazine hydrate (3.33 g, 85 %) and diethylene glycol (20 mL) was added potassium hydroxide (3.33 g). The reaction mixture was heated to 200 ^oC for 1 h in a pressure tube. The reaction mix was allowed to cool to room temperature, diluted with water and extracted with dichloromethane. Solvent was removed *in vacuo* and residual solid was recrystallized from aqueous ethanol as a pale yellow solid 1.57 g (84%), Mp. 82-84 ^oC (lit. Mp. 79-80 ^oC [3]). IR$_{Vmax}$ (ATR): 3088, 2981 (C-H), 1659 (C=C), 1555, 1478 (C=C) cm^{-1}. ^1H NMR (400 MHz, $CDCl_3$) δ 3.15 (s, 3 H, CH_3), 7.47 - 7.62 (m, 4 H, 4 x ArH), 8.03 - 8.11 (m, 2 H, 2 x ArH), 8.32 - 8.37 (m, 2 H, 2 x ArH), 8.39 (s, 1 H, H10). ^{13}C NMR (101 MHz, $CDCl_3$) 13.9 (CH_3), 124.6 (CH), 124.7 (CH), 125.2 (CH), 125.2 (CH), 129.0 (CH), 130.1, 130.1, 131.4 ppm. HRMS (APCI) calculated for $C_{15}H_{13}$ [M^++H] 193.1012: found 193.1006.

10-Ethylanthracene-9-carbaldehyde (14a)

To a cooled, stirred solution of 9-ethylanthracene (1.65 g, 8 mmol) in phosphorus oxychloride (2.30 g, 15 mmol) was added N-methylformanilide (2.03 g, 15 mmol). The flask was heated to 100 ^oC for 1.5 h. The solution was then allowed to cool to room

temperature and quenched by adding a solution of sodium acetate 8.3 g in water 15 mL. The reaction mixture was then extracted with dichloromethane, washed with water and brine and subsequently dried over sodium sulphate. The solvent was evaporated *in vacuo.* The product was purified by column chromatography (dichloromethane: hexane (1:4)), to afford a yellow solid 1.51 g (80%), Mp. 95-98 ^{o}C (lit. Mp. 96-96.5 ^{o}C [7]). IR$_{Vmax}$ (ATR): 3023, 2978 (C-H), 1603 (C=C), 1523, 1448 (C=C) cm^{-1}. ^{1}H NMR (400 MHz, $CDCl_3$) δ 1.48 (t, *J* = 7.63 Hz, 3 H, CH_3), 3.67 (q, *J* = 7.83 Hz, 2 H, CH_2), 7.48 - 7.72 (m, 4 H, 4 x ArH), 8.35 (d, *J* = 8.61 Hz, 2 H, 2 x ArH), 8.96 (d, *J* = 9.00 Hz, 2 H, 2 x ArH), 11.46 (s, 1 H, CHO). ^{13}C NMR (101 MHz, $CDCl_3$) 15.5 (CH_3), 22.1 (CH_2), 121.6 (CH), 123.5 (CH), 124.2 (CH), 125.0 (CH), 125.6 (CH), 125.7 (CH), 127.3 (CH), 128.3 (CH), 128.6, 129.0 (CH), 129.2 (CH), 131.8, 135.2 (CH), 146.0, 193.4 (CHO) ppm. HRMS (APCI) calculated for $C_{17}H_{15}O$ [M^{+}+H] 235.1123: found 235.1121.

10-Phenylanthracene-9-carbaldehyde (14c)

To a cooled, stirred solution of 9-phenylanthracene (2 g, 8 mmol) in phosphorus oxychloride (2.30 g, 15 mmol) was added N-methylformanilide (2.03 g, 15 mmol). The flask was heated to 100 ^{o}C for 1.5 h. The solution was then allowed to cool to room temperature and quenched by adding a solution of sodium acetate 8.3 g in water 15 mL. The reaction mixture was then extracted with dichloromethane, washed with water and brine and subsequently dried over sodium sulphate. The solvent was evaporated *in vacuo.* The product was purified by column chromatography (dichloromethane: hexane (1:4)) t afford a yellow solid 1.58 g (70%), Mp. 162-163 ^{o}C (lit Mp. 165-166 ^{o}C [8]). IR$_{Vmax}$ (ATR): 2981, 2884 (C-H), 1732 (C=O), 1665 (C=C), 1554, 1479 (C=C) cm^{-1}. ^{1}H NMR (400 MHz, $CDCl_3$) δ 7.32 - 7.48 (m, 4 H, 4 x ArH), 7.54 - 7.68 (m, 5 H, 5 x ArH), 7.72 (d, *J* = 8.71 Hz, 2 H, 2 x ArH), 9.01 (d, *J* = 9.12 Hz, 2 H, 2 x ArH), 11.55 (s, 1 H, CHO). ^{13}C NMR (101 MHz, $CDCl_3$) 123.3 (CH), 124.9, 125.4 (CH), 127.9 (CH), 127.9 (CH), 128.4 (CH), 128.5 (CH), 129.7, 130.5 (CH), 131.5, 138.1, 145.3, 193.1 (CHO) ppm. HRMS (APCI) calculated for $C_{21}H_{15}O$ [M^{+}+H] 283.1117: found 283.1112.

10-Methoxyanthracene-9-carbaldehyde (14d)

To a cooled, stirred solution of 9-methoxyanthracene (2 g, 8.5 mmol) in phosphorus oxychloride (2.30 g, 15 mmol) was added N-methylformanilide (2.03 g, 15 mmol). The flask was heated to 100 ^{o}C for 1.5 h. The solution was then allowed to cool to room temperature and quenched by adding a solution of sodium acetate 8.3 g in water 15 mL. The reaction mixture was then extracted with dichloromethane, washed with water and brine and subsequently dried over sodium sulphate. The solvent was evaporated *in*

vacuo. The product was recrystallized from ethanol as a bright yellow solid 1.45 g (72%), Mp. 160-162 °C (lit. Mp. 165 °C [36]). IR$_{Vmax}$ (ATR): 3029, 2997 (C-H), 1730 (C=O), 1625 (C=C), 1555, 1436 (C=C) cm^{-1}. ^{1}H NMR (400 MHz, $CDCl_3$) δ 4.21 (s, 3 H, OCH_3), 7.54 - 7.63 (m, 2 H, 2 x ArH), 7.65 - 7.75 (m, 2 H, 2 x ArH), 8.42 (d, *J* = 8.55 Hz, 2 H, 2 x ArH), 9.08 (d, *J* = 9.16 Hz, 2 H, 2 x ArH), 11.48 (s, 1 H, CHO). ^{13}C NMR (101 MHz, $CDCl_3$) 63.9 (CH_3), 121.2 (CH), 123.1 (CH), 123.9 (CH), 124.2, 125.6 (CH), 127.0 (CH), 127.2 (CH), 128.4, 129.3 (CH), 133.8, 134.1 (CH), 159.2 (C10), 191.9 ppm (CHO). HRMS (APCI) calculated for $C_{16}H_{13}O_2$ [M^++H] 237.0916: found 237.0907.

10-Methylanthracene-9-carbaldehyde (14e)

To a cooled, stirred solution of 9-methylanthracene (2 g, 8.5 mmol) in phosphorus oxychloride (2.30 g, 15 mmol) was added N-methylformanilide (2.03 g, 15 mmol). The flask was heated to 100 °C for 1.5 h. The solution was then allowed to cool to room temperature and quenched by adding a solution of sodium acetate 8.3 g in water 15 mL. The reaction mixture was then extracted with dichloromethane, washed with water and brine and subsequently dried over sodium sulphate. The solvent was evaporated *in vacuo*. The product was recrystallized from ethanol as a bright orange solid 1.12 g (60%), Mp. 171-174 °C (lit. Mp. 171.9-172.6 °C [9]). IR$_{Vmax}$ (KBr): 3076, 2981 (C-H), 1681 (C=O), 1561 (C=C), 1542, 1449 (C=C) cm^{-1}. ^{1}H NMR (400 MHz, $CDCl_3$) δ 3.18 (s, 3 H, CH_3), 7.51 - 7.77 (m, 4 H, 4 x ArH), 8.39 (d, *J* = 9.16 Hz, 2 H, 2 x ArH), 8.99 (d, *J* = 8.55 Hz, 2 H, 2 x ArH), 11.50 (s, 1 H, CHO). ^{13}C NMR (101 MHz, $CDCl_3$) 15.4 (CH_3), 123.9, 124.1 (CH), 125.4 (CH), 125.6 (CH), 128.4 (CH), 129.7, 131.6, 139.9, 193.4 (CHO) ppm HRMS (APCI) calculated for $C_{16}H_{13}O$ [M^++H] 221.0961: found 221.0953.

10-Bromoanthracene-9-carbaldehyde (14f)

To a suspension of 9,10-dibromoanthracene (2 g, 5.95 mmol) in dry THF (20 mL) under nitrogen, at -90 °C was added *n*-butyl lithium (2.9 mL, 7.2 mmol, 2.5 M in hexane) dropwise. The solution was stirred at -90 °C for 50 min before the addition of N-methylformanilide (1.46 mL, 11.9 mmol). The reaction mixture was allowed to heat to room temperature and was stirred overnight. The reaction was quenched with the addition of deionised water (50 mL) and extracted into dichloromethane. Organic layers were combined, washed with water and brine and dried over sodium sulphate. The solvent was removed *in vacuo* and the residue was purified by column chromatography (dichloromethane: hexane (1:2)) to afford a yellow solid (72%), Mp. 210-213 °C (lit.

Mp. 218 ^{o}C [11]). IR$_{Vmax}$ (ATR): 3086, 2970 (C-H), 1731 (C=O), 1666 (C=C), 1544, 1439 (C=C) cm^{-1}. ^{1}H NMR (400 MHz, $CDCl_3$) δ 7.59 - 7.74 (m, 4 H, 4 x ArH), 8.61 - 8.68 (m, 2 H, 2 x ArH), 8.79 - 8.90 (m, 2 H, 2 x ArH), 11.47 (s, 1 H, CHO). ^{13}C NMR (101 MHz, $CDCl_3$) 123.8, 125.6 (CH), 127.3 (CH), 128.8 (CH), 128.9 (CH), 130.2, 131.8, 131.8, 193.2 (CHO) ppm. HRMS (APCI) calculated for $C_{15}H_{10}Br^{79}O$ [M^{+}+H] 284.9915: found 284.9906.

4,5-Dichloroanthracene-9-carbaldehyde (14g)

To a solution of 1,8-dichloroanthracene (5 g, 20.3 mmol) and $AlCl_3$ (5 g in dichloromethane (145 mL) was added of dichloromethyl methyl ether (2.7 g in dichloromethane 2 mL). After 1 h the reaction was poured onto ice cold dilute HCl and extracted into dichloromethane. The organic extracts were combined, washed with water and brine and dried over sodium sulphate. Solvent was removed *in vacuo*. Purification was carried out using column chromatography (eluent: toluene) to afford the product as a yellow solid 1.67 g (30%), Mp. 220-222 ^{o}C (lit. Mp. 224-226 ^{o}C [13]). IR$_{Vmax}$ (ATR): 3094, 2981 (C-H), 1734 (C=O), 1618 (C=C), 1510, 1479 (Ar C=C) cm^{-1}. ^{1}H NMR (400 MHz, $CDCl_3$) δ 7.54 - 7.64 (m, 2 H, 2 x ArH), 7.64 - 7.73 (m, 2 H, 2 x ArH), 8.77 (d, *J* = 8.29 Hz, 2 H, 2 x ArH), 9.58 (br. s., 1 H, H10), 11.41 (br. s., 1 H, CHO). ^{13}C NMR (101 MHz, $CDCl_3$) 122.3 (CH), 126.1 (CH), 128.1 (CH), 128.5 (CH), 128.6 (CH), 130.4, 132.2, 133.2, 192.5 (CHO) ppm. HRMS (APCI) calculated for $C_{15}H_9Cl_2{}^{35}O$ [M^{+}+H] 275.0030: found 275.0030.

9,10-Dibromoanthracene (15)

To a solution of anthracene (5 g, 28 mmol) in dichloromethane (100 mL) was added dropwise bromine (2.9 mL) in dichloromethane (50 mL). The reaction mixture was allowed to stir at room temperature for 4 h. The solvent was removed *in vacuo* and residual solid was recrystallized from dichloromethane as a yellow solid, 8.41 g (90%), Mp. 210-215 ^{o}C (lit. Mp. 221 ^{o}C [10]). IR$_{Vmax}$ (ATR): 3077, 2981, (C-H), 1621 (C=C), 1506, 1435 (C=C) cm^{-1}. ^{1}H NMR (400 MHz, $CDCl_3$) δ 7.57 - 7.71 (m, 4 H, 4 x ArH), 8.48 - 8.67 (m, 4 H, 4 x ArH). ^{13}C NMR (101 MHz, $CDCl_3$) 123.5, 127.5 (CH), 128.3 (CH), 131.1 ppm. HRMS (APCI) calculated for $C_{14}H_9Br_2{}^{79}$ [M^{+}+H] 334.9071: found 334.9084.

1,8-Dichloroanthracene (16)

A slurry of 1,8-dichloroanthracene-9,10-dione (5 g, 18.1 mmol) in NH_3 (60 mL , aqueous 28 %) and deionised water (45 mL) was cooled in an ice bath. Zinc dust (25 g,

0.38 mol) was added portion-wise with stirring over 15 min. The resulting red coloured slurry was heated slowly to 75 °C and stirred at this temperature for 4 h. The reaction mixture was allowed to cool to room temperature and was filtered to remove excess zinc solids. The filtrate was extracted with dichloromethane and the organic extracts were concentrated *in vacuo* to give a white residue. This residual white solid was suspended in a mixture of 12 M HCl (20 mL) and isopropanol (220 mL). The resulting suspension was refluxed for 3 h resulting in a pale yellow solution. The reaction mixture was allowed to stand overnight at room temperature and the precipitated solid was collected by filtration. Further concentration of the mother liquor led to the formation of a second crop of crystals when allowed to stand for a further 24 h. The product was isolated as yellow crystals 2.53 g (57%), Mp. 152-155 °C (lit. Mp. 156-157 °C [12]). IR_{Vmax} (ATR): 3028, 2981 (C-H), 1618 (C=C), 1508, 1382 (C=C) cm^{-1}. ^{1}H NMR (400 MHz, $CDCl_3$) δ 7.36 - 7.42 (m, 2 H, 2 x ArH), 7.59 - 7.64 (m, 2 H, 2 x ArH), 7.90 (d, *J* = 8.71 Hz, 2 H, 2 x ArH), 8.40 (s, 1 H, H10), 9.21 (s, 1 H, H9). ^{13}C NMR (101 MHz, $CDCl_3$) 120.9 (CH), 125.6 (CH), 125.9 (CH), 127.2 (CH), 127.5 (CH), 129.4, 132.4, 132.5 ppm. HRMS (APCI) calculated for $C_{14}H_9Cl_2^{35}$ [M^+] 246.0003: found 245.9999.

2-(Anthracen-9-yl)acetaldehyde (18)

To a solution of (1.8 g, 7.6 mmol) of the enolether **208** and NaI (13 mmol) in dry acetonitrile (76 mL) under nitrogen was added TMSCI (13 mmol) dropwise. The reaction was stirred vigorously for 10 min and poured over of 0.5 M aqueous sodium thiosulfate 100 mL and extracted into diethyl ether. The combined organic layers were washed with water, brine and subsequently dried over sodium sulphate. The solvent was removed *in vacuo*. The product was purified by column chromatography (hexane: dichloromethane (3:2)) to afford a yellow solid 1.39 g (83%), Mp. 140-144 °C (lit. Mp. 144-145 °C [14]). IR_{Vmax} (ATR): 3032, 2881 (C-H), 1621 (C=C), 1522, 1423 (C=C) cm^{-1}. ^{1}H NMR (400 MHz, $CDCl_3$) δ ppm 4.67 (d, *J* = 1.83 Hz, 2 H, CH_2), 7.46 - 7.62 (m, 4 H, 4 x ArH), 8.07 (d, *J* = 8.55 Hz, 2 H, 2 x ArH), 8.18 (d, *J* = 9.16 Hz, 2 H, 2 x ArH), 8.48 (s, 1 H, H10), 9.76 - 9.82 (m, 1 H, CHO). ^{13}C NMR (101 MHz, $CDCl_3$) 43.0 (CH_2), 123.3, 123.7 (CH), 125.1 (CH), 126.5 (CH), 127.7 (CH), 129.3 (CH), 131.4, 198.8 (CHO) ppm. HRMS (APCI) calculated for $C_{16}H_{13}O$ [M^++H] 221.0966: found 221.0960.

General procedure for the preparation of nitrovinyl anthracene derivatives (19a-g, 21)

To a solution of 9-anthraldehyde (2 g, 9.7 mmol) in the appropriate nitroalkane (15 mL) was added piperidinium acetate (1.5 g, 10.3 mmol). (Piperidinium acetate was prepared from piperidine 6.6 mL and acetic acid 3 mL). The solution was heated at 90 ^{o}C for 1.5 h under nitrogen. After one hour the reaction was cooled to room temperature and poured onto 100 mL of ice cold H_2O. The resultant mixture was extracted into DCM, washed with brine and the organic layers were combined, dried over Na_2SO_4 and solvent removed *in vacuo*. The product was recrystallised from an appropriate solvent.

(*E*)-9-(2-nitrovinyl)anthracene (19a)

(*E*)-9-(2-Nitrovinyl)anthracene was prepared from 9-anthraldehyde (9.7 mmol, 2 g) and nitromethane (15 mL) according to general procedure above. The product was recrystallized from methanol and diethyl ether as red crystals, 2.41 g (99%), Mp. 145-147 ^{o}C (lit. Mp. 142 ^{o}C [15]). IR$_{Vmax}$ (KBr): 3050, 2948 (C-H), 1617, 1553 (C=C), 1498, 1330 (NO_2), 1250 (C-N) cm^{-1}. ^{1}H NMR (400 MHz, $CDCl_3$) δ 7.40 - 7.66 (m, 5 H, 4 x ArH, CH= x 1), 7.80 - 8.05 (m, 2 H, 2 x ArH), 8.05 - 8.25 (m, 2 H, 2 x ArH), 8.45 (br. s., 1 H, H10), 8.90 (d, *J* = 13.43 Hz, 1 H, CH= x 1). ^{13}C NMR (101 MHz, $CDCl_3$) 124.3 (C9) , 125.7 (CH) , 127.5 (CH) , 129.2 (CH) , 129.2, 129.8, 130.4 (CH) , 131.1, 135.6 (CH) , 142.6 (C2') ppm. HRMS (APCI) calculated for $C_{16}H_{12}NO_2$ [M^{+}+H] 250.0868: found 250.0879.

(*E*)-9-(2-Nitroprop-1-en-1-yl)anthracene (19b)

(*E*)-9-(2-Nitroprop-1-en-1-yl)anthracene was prepared from 9-anthraldehyde (9.7 mmol, 2 g) and nitroethane (15 mL) according to general procedure above. The product was recrystallized from ethanol and diethyl ether as orange crystals 1.87 g (73%), Mp. 141-142 ^{o}C (lit. Mp. 142-143 ^{o}C [16]). IR$_{Vmax}$ (KBr): 3052, 2825 (C-H), 1510, 1326 (NO_2), 1622.16, 1442.74 (C=C), 1483 (C=C), 1385 (CH_3) cm^{-1}. ^{1}H NMR (400 MHz, $CDCl_3$) δ 1.98 (s, 1 H, CH_3), 7.42 - 7.63 (m, 4 H, 4 x ArH), 7.86 (d, *J* = 7.93 Hz, 2 H, 2 x ArH), 7.93 - 8.12 (m, 2 H, 2 x ArH), 8.48 (s, 1 H, H10), 8.71 (s, 1 H, H1'). ^{13}C NMR (101 MHz, $CDCl_3$) 14.4 (C3'), 124.8 (C9), 125.5 (CH), 126.7, 128.6, 128.8 (CH), 129.0 (CH), 131.0, 131.3 (CH), 151.0 (C2') ppm. HRMS (ESI) calculated for $C_{17}H_{14}NO_2$ [M^{+}+H] 264.1025: found 264.1035.

(*E*)-9-(2-Nitrobut-1-en-1-yl)anthracene (19c)

(*E*)-9-(2-Nitrobut-1-en-1-yl)anthracene was prepared from 9-anthraldehyde (9.7 mmol, 2 g) and nitropropane (15 mL) according to general procedure above. The product was

recrystallized from ethanol and diethyl ether as gold crystals 1.62 g (60%), Mp: 159-160 °C [17]. IR$_{Vmax}$ (KBr): 2982, 2937 C-H), 1622, 1427 (C=C), 1520, 1341 (NO_2), 1444 (CH_2), 1376 (CH_3) cm^{-1}. ^{1}H NMR (400 MHz, $CDCl_3$) δ 0.90 (t, *J* = 7.32 Hz, 3 H, CH_3), 2.37 (q, *J* = 7.32 Hz, 2 H, CH2), 7.48 - 7.54 (m, 4 H,4 x ArH), 7.88 - 7.93 (m, 2 H, H4, H5), 8.01 - 8.05 (m, 2 H, H8, H1), 8.48 (s, 1 H, H10), 8.54 (s, 1 H, H1'). ^{13}C NMR (101 MHz, $CDCl_3$) 11.6 (C3'), 21.4 (C2'), 124.9 (C9), 125.6 (CH), 125.7, 126.6 (CH), 128.3 (CH), 128.8, 128.9 (CH), 130.7 (CH), 131.1, 156.7 (C2') ppm. HRMS (APCI) calculated for $C_{18}H_{16}NO_2$ [M^++H] 278.1181: found 278.1189.

(*E*)-9-Chloro-10-(2-nitrovinyl)anthracene (19d)

(*E*)-9-Chloro-10-(2-nitrovinyl) anthracene was prepared from 10-chloroanthracene-9-carbaldehyde (5 mmol, 1.2 g) and nitromethane (15 mL) according to general procedure above. The product was recrystallized from methanol and diethyl ether as orange crystals, 1.01 g (71%), Mp. 232-234 °C [34]. IR$_{Vmax}$ (ATR): 3066, 2973 (C-H), 1623 (C=C), 1439 (C=C), 1538, 1326 (NO_2), 1110 (C-N) cm^{-1}. ^{1}H NMR (400 MHz, $CDCl_3$) δ 7.52 (d, *J* = 14.04 Hz, 1 H, =CH), 7.60 - 7.77 (m, 4 H, 4 x ArH), 8.20 (d, *J* = 8.55 Hz, 2 H, 2 x ArH), 8.62 (d, *J* = 8.55 Hz, 2 H, 2 x ArH), 8.96 (d, *J* = 14.04 Hz, 1 H, =CH). ^{13}C NMR (101 MHz, $CDCl_3$) 124.8, 125.8, 127.1, 127.6, 128.5, 130.1, 135.4 (C1), 143.3 (C2) ppm. HRMS (APCI) calculated for $C_{16}H_{11}Cl^{35}NO_2$ [M+H] 284.0478: found 284.0492.

(*E*)-9-Chloro-10-(2-nitroprop-1-en-1-yl)anthracene (19e)

(*E*)-9-Chloro-10-(2-nitroprop-1-en-1-yl)anthracene was prepared from 10-chloroanthracene-9-carbaldehyde (5 mmol, 1.2 g) and nitroethane (15 mL) according to general procedure above. The product was recrystallized from methanol and diethyl ether as orange crystals, 722 mg (50%), Mp. 158-160 °C [34]. IR$_{Vmax}$ (ATR): 3023, 2977 (C-H), 1622 (C=C), 1480, 1438 (C=C), 1512, 1327 (NO_2), 1171 (C-N) cm^{-1}. ^{1}H NMR (400 MHz, $CDCl_3$) δ 1.99 (s, 3 H, CH_3), 7.53 - 7.77 (m, 4 H, 4 x ArH), 7.92 (d, *J* = 8.55 Hz, 2 H, 2 x ArH), 8.60 (d, *J* = 9.16 Hz, 2 H, 2 x ArH), 8.70 (s, 1 H, H1'). ^{13}C NMR (101 MHz, $CDCl_3$) 14.4 (C3'), 125.2, 125.6, 127.0, 127.0, 128.4, 129.2, 130.8, 131.0 (C1'), 151.5 (C2') ppm. HRMS (APCI) calculated for $C_{17}H_{13}Cl^{35}NO_2$ [M^++H] 298.0635: found 298.0637.

(*E*)-9-Chloro-10-(2-nitrobut-1-en-1-yl)anthracene (19f)

(*E*)-9-Chloro-10-(2-nitrobut-1-en-1-yl)anthracene was prepared from 10-chloroanthracene-9-carbaldehyde (5 mmol, 1.2 g) and nitropropane (15 mL) according to general procedure above. The product was recrystallized from methanol and diethyl

ether as orange crystals, 998 mg (64%), Mp. 157-159 °C. [34] IR$_{Vmax}$ (ATR): 3086, 2850 (C-H), 1621 (C=C), 1438, 1425 (C=C), 1553, 1328 (NO_2), 1149 (C-N) cm^{-1}. ^{1}H NMR (400 MHz, $CDCl_3$) δ 0.90 (t, *J* = 7.32 Hz, 3 H, CH_3), 2.35 (q, *J* = 7.32 Hz, 2 H, CH_2), 7.52 - 7.70 (m, 4 H, 4 x ArH), 7.94 (d, *J* = 8.55 Hz, 2 H, H4, H6), 8.49 (s, 1 H, H1'), 8.57 (d, *J* = 8.55 Hz, 2 H, H1 & H8). ^{13}C NMR (101 MHz, $CDCl_3$) 11.5 (C4'), 21.4 (C3') 125.3 (CH), 125.2 (CH), 125.4 (CH), 125.5, 126.9 (CH), 128.4, 129.1, 130.1 (C1'), 130.6, 157.0 (C2') ppm. HRMS (APCI) calculated for $C_{18}H_{15}Cl^{35}NO_2$ [M^++H] 312.0791: found 312.0797.

(*E*)-9-Methoxy-10-(2-nitrovinyl)anthracene (19g)

(*E*)-9-Methoxy-10-(2-nitrovinyl)anthracene was prepared from 10-methoxy anthracene-9-carbaldehyde (2 mmol, 0.5 g) and nitromethane (6 mL) according to general procedure above. The product was purified by column chromatography (dichloromethane: hexane (1:1)). The product was recrystallized from methanol and diethyl ether as an orange solid 302 mg (54%), Mp. 151-153 °C [18]. IR$_{Vmax}$ (KBr): 3048, 2971 (C-H), 1610, 1468 (C=C), 1559, 1352 (NO_2), 1234 (C-N) cm^{-1}. ^{1}H NMR (400 MHz, $CDCl_3$) δ 4.19 (s, 3 H, OCH_3), 7.53 - 7.66 (m, 5 H, 4 x ArH, H1'), 8.24 (d, *J* = 9.16 Hz, 2 H, 2 x ArH), 8.40 (d, *J* = 7.94 Hz, 2 H, 2 x ArH), 9.02 (d, *J* = 13.43 Hz, 1 H, H2'). ^{13}C NMR (101 MHz, $CDCl_3$) 63.7 (OCH_3), 123.1 (CH), 124.3, 124.7 (CH), 125.6 (CH), 127.7 (CH), 131.1, 135.5 (CH), 142.3 (CH), 155.4 (C2') ppm. HRMS (APCI) calculated for $C_{17}H_{14}NO_3$ [M^++H] 280.0974: found 280.0965.

(*E*)-9-(2-Nitrovinyl)phenanthrene (21)

(*E*)-9-(2-Nitrovinyl)phenanthrene was prepared from phenanthrene-9-carbaldehyde (7.3 mmol, 1.5 g) and nitromethane (18 mL) according to general procedure above. The product was purified by column chromatography (eluent: toluene) and recrystallized from ethanol as an orange solid 273 mg (15%), Mp. 170-173 °C (lit. Mp. 173 °C [19]. IR $_{Vmax}$ (ATR): 2981, 2885 (C-H), 1633 (C=C), 1534, 1338 (NO_2), 1442 (C=C) cm^{-1}. ^{1}H NMR (400 MHz, $CDCl_3$) δ 7.65 - 7.78 (m, 5 H, 4 x ArH & H1'), 7.91 (d, J = 7.94 Hz, 1 H, 1 x ArH), 7.95 (s, 1 H, H10), 8.11 (d, J = 8.55 Hz, 1 H, 1 x ArH), 8.68 (d, J = 8.55 Hz, 1 H, 1 x ArH), 8.74 (d, J = 7.93 Hz, 1 H, 1 x ArH), 8.79 (d, J = 13.43 Hz, 1 H, H2'). ^{13}C NMR (101 MHz, $CDCl_3$) 122.7 (CH), 122.8, 123.4 (CH), 123.9 (CH), 126.1, 127.4 (CH), 127.5 (CH), 127.5 (CH), 128.4 (CH), 128.7 (CH), 129.5, 129.5 (CH), 130.6, 131.7, 136.8 (CH), 138.8 (C2') ppm. HRMS (APCI) calculated for $C_{16}H_{12}NO_2$ [M^++H] 250.0868: found 250.0861.

4-(Anthracen-9-yl)-1*H*-1,2,3-triazole (30a)

To a stirred solution of (*E*)-9-(2-nitrovinyl)anthracene (1 mmol, 0.25 g) in DMSO (2 mL) was added sodium azide (2 mmol). The reaction was stirred at 90 ^{o}C for 90 min. After allowing the reaction to cool to room temperature, deionised water (10 mL) was added and the aqueous mixture was extracted into diethyl ether. The organic layer was washed with water, brine and dried over sodium sulphate. Solvent was removed *in vacuo* to afford crude product. The product was purified by column chromatography (gradient dichloromethane – methanol) and recrystallized from ethanol to afford a yellow solid, 226 mg (92%), Mp. 241-245 ^{o}C (lit. Mp. 243-244 ^{o}C [20]). IR$_{Vmax}$ (KBr): 3035, 2881 (C-H), 1624. (C=C), 1554, 1441 (C=C), 1149 (C-N) cm^{-1}. ^{1}H NMR (400 MHz, DMSO-*d*$_6$) δ 7.43 - 7.59 (m, 4 H, 4 x ArH), 7.68 (d, *J* = 9.16 Hz, 2 H, 2 x ArH), 8.16 (d, *J* = 8.55 Hz, 2 H, 2 x ArH), 8.23 (s, 1 H, H10), 8.74 (s, 1 H, =CH). ^{13}C NMR (101 MHz, DMSO-*d*$_6$) 125.5 (CH), 125.6 (CH), 126.4 (CH), 128.0 (CH), 128.5 (CH), 130.6, 130.8. ppm. HRMS (APCI) calculated for $C_{16}H_{12}N_3$ [M$^+$+H] 246.1031: found 246.1033.

(*E*)-(Anthracen-9-ylmethylene)hydrazine (32a)

To a solution of 9-anthraldehyde (1 g, 4.85 mmol) in DCM (20 ml) and ethanol (10 mL), was added dropwise hydrazine (1 g, 20 mmol). The solution was then stirred at room temperature for 24 h. The solvent was removed *in vacuo*. The residual solid was recrystallized from a mixture of dichloromethane and hexane. The product was isolated as an orange solid, 0.965 g (90%), Mp. 122-124 ^{o}C (lit. Mp. 125-126 ^{o}C [21]). IR$_{Vmax}$ (ATR): 3363 (N-H), 3044, 2982 (C-H), 1622 (C=C), 1484, 1440 (C=C) cm^{-1}. ^{1}H NMR (400 MHz, DMSO-*d*$_6$) δ 7.18 (s, 2 H, NH$_2$), 7.52 (br. s., 4 H, 4 x ArH), 8.07 (d, *J* = 7.32 Hz, 2 H, 2 x ArH), 8.52 (s, 1 H, C10), 8.62 (d, *J* = 8.55 Hz, 2 H, 2 x ArH), 8.89 (s, 1 H, C1'). ^{13}C NMR (101 MHz, DMSO-*d*$_6$) 125.3 (CH), 125.4 (CH), 125.9 (CH), 126.8 (CH), 127.8, 128.6 (CH), 129.0, 131.1, 136.5 (C1') ppm. HRMS (APCI) calculated for $C_{15}H_{13}N_2$ [M$^+$+H] 221.1079: found 221.1080.

(*E*)-((10-Chloroanthracen-9-yl)methylene)hydrazine (32b)

To a solution of 10-chloro-9-anthraldehyde (1.16 g, 4.85 mmol) in DCM (20 mL) and ethanol (10 mL), was added dropwise hydrazine (1 g, 20 mmol). The solution was then stirred at RT for 24 h. The solvent was removed *in vacuo*. The residual solid was recrystallized from a mixture of dichloromethane and hexane. The product was isolated by filtration, and obtained as an orange solid 1.05 g (85%), Mp. 205-207 ^{o}C.[34] IR$_{Vmax}$ (KBr): 3351 (N-H), 3038, 2904 (C-H), 1621 (C=C), 1483, 1438 (C=C) cm^{-1}. ^{1}H NMR (400 MHz, DMSO-*d*$_6$) δ 7.30 (s, 2 H, NH$_2$), 7.58 - 7.67 (m, 2 H, 2 x ArH), 7.67 - 7.76 (m, 2 H, 2 x ArH), 8.45 (d, *J* = 8.55 Hz, 2 H, 2 x ArH), 8.63 (d, *J* =

8.55 Hz, 2 H, 2 x ArH), 8.82 (s, 1 H, C1'). ^{13}C NMR (101 MHz, DMSO-d_6) 109.6 (C10), 124.3 (CH), 126.2 (CH), 126.3 (CH), 127.0, 127.4 (CH), 127.9, 128.7, 129.4, 135.5 (C1') ppm. HRMS (APCI) calculated for $C_{15}H_{12}N_2Cl^{35}$ [M$^+$+H] 255.0689: found 255.0684.

(*E*)-Anthracene-9-carbaldehyde oxime (32c)

9-Anthraldehyde (1 g) was dissolved in ethanol (20 mL) and heated to 75 ^oC. To this was added hydroxylamine hydrochloride (0.4 g) in water (3.33 mL), neutralised with sodium carbonate. The mixture was heated for ten minutes and diluted with water until cloudy. The reaction mixture was then cooled on ice and the precipitate was filtered to give the product as a white solid 0.99 g (90%), Mp. 157-160 ^oC (lit. Mp. 159-162 ^oC [22]). IR$_{Vmax}$ (ATR): 3420 (O-H), 3084, 3012, 2980 (C-H), 1634, 1622, 1482 (C=C) cm^{-1}. ^1H NMR (400 MHz, DMSO-d_6) δ 7.45 - 7.65 (m, 4 H, 4 x ArH), 8.03 - 8.19 (m, 2 H, 2 x ArH), 8.41 - 8.54 (m, 2 H, 2 x ArH), 8.62 (s, 1 H, H10), 9.23 (s, 1 H, OH). ^{13}C NMR (101 MHz, DMSO-d_6) 124.8, 125.1 (CH), 125.2 (CH), 125.4 (CH), 126.1 (CH), 126.7 (CH), 128.4 (CH), 128.6, 128.7 (CH), 129.5, 130.9, 146.4 (CH), 146.5 (CH) ppm. HRMS (APCI) calculated for $C_{15}H_{12}NO$ [M$^+$+H] 222.0919: found 222.0914.

(*E*)-1-(Anthracen-9-yl)-N-methylmethanimine oxide (32d)

To an ice-cold solution of sodium acetate (7.5 mmol) in dichloromethane (30 mL), was added N-methylhydroxylamine (7.5 mmol). A solution of 9-anthaldehyde (1 g, 5 mmol) in dichloromethane was added dropwise. The reaction mixture was stirred at 0 ^oC for 2 h and allowed to warm to room temperature overnight. The organic layer was washed successively with brine, water and dried over sodium sulphate. The solvent was removed *in vacuo* and the residual solid was recrystallized from ethanol and diethyl ether as colourless crystals, 1.05 g (89%), Mp. 90-93 ^oC (lit. Mp. 92-94 ^oC [23]). IR$_{Vmax}$ (ATR): 2981, 2895 (C-H), 1622, 1484, 1438 (C=C), 1146 (C-N) cm^{-1}. ^1H NMR (400 MHz, $CDCl_3$) δ 4.15 (s, 3 H, CH_3), 7.44 - 7.59 (m, 4 H, 4 x ArH), 7.87 (d, J = 8.55 Hz, 2 H, 2 x ArH), 8.01 (d, J = 7.93 Hz, 2 H, 2 x ArH), 8.25 (s, 1 H, C10), 8.51 (s, 1 H, C1'). ^{13}C NMR (101 MHz, $CDCl_3$) 53.7 (CH_3), 122.3, 124.3 (CH), 125.0 (CH), 125.4 (CH), 125.8 (CH), 126.7 (CH), 127.6 (CH), 129.0 (CH), 129.1 (CH), 129.5, 130.0 (CH), 131.2, 134.1 (C1') ppm. HRMS (APCI) calculated for $C_{16}H_{14}NO$ [M$^+$+H] 236.1066: found 236.1070.

(*E*)-2-(Anthracen-9-yl)acetaldehyde oxime (32e)

9-Anthraldehyde (1 g) was dissolved in ethanol (20 mL) and heated to 75 ^{o}C. To this solution was added hydroxylamine hydrochloride (0.4 g) in water (3.33 mL) neutralised with sodium carbonate. The mixture was heated for ten minutes and diluted with water until cloudy. The reaction mixture was then cooled on ice and the precipitate was filtered to give the product as yellow crystals, 236 mg (20%), Mp. 178-180 ^{o}C (lit. Mp. 177-180 ^{o}C [24]). IR$_{Vmax}$ (ATR): 3025, 2852 (C-H), 1621, 1488 (C=C) cm^{-1}. ^{1}H NMR (400 MHz, DMSO-d_6) δ 4.63 (d, *J* = 5.49 Hz, 2 H, CH_2), 6.68 (t, *J* = 5.19 Hz, 1 H, CH), 7.51 - 7.61 (m, 4 H, 4 x ArH), 8.12 (d, *J* = 7.94 Hz, 2 H, 2 x ArH), 8.31 (d, *J* = 8.55 Hz, 2 H, 2 x ArH), 8.57 (s, 1 H, H10), 11.39 (s, 1 H, =N-OH). ^{13}C NMR (101 MHz, DMSO-d_6) 24.0 (CH_2), 124.3 (CH), 125.2 (CH), 126.2 (CH), 126.3 (CH), 129.0 (CH), 129.3, 129.7, 131.1, 147.9 (N=CH) ppm. HRMS (APCI) calculated for $C_{16}H_{14}NO$ [M^{+}+H] 236.1075: found 236.1069.

2-(Anthracen-9-ylmethylene)malononitrile (23a)

To a solution of 9-anthraldehyde (0.393 g, 1.91 mmol) and malononitrile (0.372 g, 6.11 mmol) in ACN (50 mL), was added 1 drop of piperidine. The solution was then heated at 90 ^{o}C for 0.5 h. The solution was then concentrated *in vacuo*, the residual solid dissolved in DCM and washed with HCl, water and brine. The organic phases were combined and dried over Na_2SO_4, solvent removed *in vacuo* and recrystallized from ethanol as orange crystals 459 mg (95%), Mp. 256-257 ^{o}C (lit. Mp. 206 ^{o}C [25]). IR$_{Vmax}$ (KBr): 2965, 2932 (C-H), 2228 (CN), 1621 (C=C), 1574, 1445 (Ar C=C) cm^{-1}. ^{1}H NMR (400 MHz, $CDCl_3$) δ 7.55 (t, *J* = 7.63 Hz, 3 H, 3 xArH), 7.65 (t, *J* = 7.63 Hz, 2 H, 2 x ArH), 7.89 (d, *J* = 8.54 Hz, 2 H, H4 & H5), 8.05 (d, *J* = 7.93 Hz, 2 H, H8 & H1), 8.59 (s, 1 H, H10), 8.88 (s, 1 H, H1'). ^{13}C NMR (101 MHz, $CDCl_3$) 92.2, 108.9, 111.5 (CN), 113.0 (CN), 123.4 (C9), 123.8, 126.1, 128.3, 129.1, 129.5, 130.8, 132.5, 160.8 (C2') ppm. HRMS (ESI) calculated for $C_{18}H_9N_2$ [M^{+}-H] 253.0771: found 253.0764.

(*E*)-3-(Anthracen-9-yl)acrylonitrile (23c)

To a solution of 9-anthraldehyde (1 g, 4.85 mmol) and cyanoacetic acid (0.52 g, 6.11 mmol) in DMF (6 mL), was added morpholine (0.7 mL). The solution was then heated at 90 ^{o}C for 6 h. The solution was then cooled to -10 ^{o}C overnight. The resultant crystals were filtered and washed with diethyl ether. The product was obtained as orange crystals 446 mg (40%), Mp. 205-207 ^{o}C (lit. Mp. 209.5-210.5 ^{o}C [26]). IR$_{Vmax}$ (KBr): 3051 (C-H), 2218 (CN), 1623, 1442.02 (C=C) cm^{-1}. ^{1}H NMR (400 MHz, $CDCl_3$) δ 5.84 (d, *J* = 17.09 Hz, 1 H =CH), 7.43 - 7.54 (m, 4 H, 4 x ArH), 7.97 (d, *J* = 7.93 Hz, 2 H, H4 & H5), 8.06 (d, *J* = 8.54 Hz, 2 H, H1 & H8), 8.27 (d, *J* = 17.09 Hz, 1 H, =CH), 8.41 (s, 1

H, H10). ^{13}C NMR (101 MHz, $CDCl_3$) 105.4 (C2'), 117.4 (CN), 124.3 (C9), 125.4 (CH), 126.9 (CH), 127.6, 128.9 (CH), 129.0 (CH), 129.2, 131.0, 148.4 (C1') ppm. HRMS (APCI) calculated for $C_{17}H_{12}N$ [M^++H] 230.0966: found 230.097.

5-(Anthracen-9-ylmethylene)-2,2-dimethyl-1,3-dioxane-4,6-dione (24)

A solution of 9-anthraldehyde (0.5 g, 2.4 mmol) and Meldrums acid (0.35 g, 2.5 mmol) in pyridine (2 mL) was stirred at room temperature for six hours. Solvent was removed from the resulting mixture *in vacuo*. The crude product was recrystallized from ethanol as an orange solid 558 mg (70%), Mp. 187-188 °C (lit. Mp. 193-194 °C [28]). IR$_{Vmax}$ (ATR): 3029, 2970 (C-H), 1730 (C=O), 1625 (C=C), 1555, 1444 (C=C) cm^{-1}. ^{1}H NMR (400 MHz, $CDCl_3$) δ 1.90 (s, 6 H, 2 x CH_3), 7.45 - 7.57 (m, 4 H, 4 x ArH), 7.83 (d, *J* = 9.16 Hz, 2 H, 2 x ArH), 7.98 - 8.07 (m, 2 H, 2 x ArH), 8.53 (s, 1 H, H10), 9.45 (s, 1 H, =CH). ^{13}C NMR (101 MHz, DMSO-d_6) 28.1 (CH_3), 104.9, 120.9, 124.4 (CH), 125.5 (CH), 126.7, 127.0 (CH), 128.5, 129.3 (CH), 130.2 (CH), 130.8, 157.6 (CH), 157.8 (C=O), 161.8 (C=O) ppm. HRMS (APCI) calculated for $C_{21}H_{16}O_4$ [M^++H] 332.1049: found 332.1051.

(*E*)-4-(Anthracen-9-yl)but-3-en-2-one (25)

To a stirred solution of 9-anthraldehyde (1 g, 4.5 mmol) in acetone (50 mL) was added aqueous 10 % NaOH 0.5 mL. The solution was stirred for 3 h and concentrated *in vacuo*. The product was isolated by filtration and recrystallized from ethanol as an orange solid 666 mg (60%), Mp. 99-100 °C (lit. Mp. 100-102 °C [27]). IR $_{Vmax}$ (ATR): 3046, 2971 (C-H), 1661 (C=O), 1622 (C=C), 1442, 1441 (C=C) cm^{-1}. ^{1}H NMR (400 MHz, $CDCl_3$) δ ppm 2.56 (s, 3 H, CH_3), 6.71 (d, *J* = 16.48 Hz, 1 H, H3'), 7.51 (m, 4 H, 4 x ArH), 8.01 (d, *J* = 7.32 Hz, 2 H, 2 x ArH), 8.20 (d, *J* = 7.32 Hz, 2 H, 2 x ArH), 8.39 - 8.53 (m, 2 H, 1 x H10, H4'). ^{13}C NMR (101 MHz, $CDCl_3$) 27.9 (CH_3), 125.0 (CH), 125.3 (CH), 126.3 (CH), 128.4 (CH), 128.9 (CH), 129.1, 129.3, 131.2, 135.8 (CH), 140.4 (CH), 197.8 (C=O) ppm. HRMS (APCI) calculated for $C_{18}H_{15}O$ [M^++H] 247.1123: found 247.1116.

Anthracen-9-ylmethanol (26)

To a suspension of 9-anthraldehyde (206 mg, 1 mmol) in ethanol (3 mL) was added sodium borohydride (152 mg, 4 mmol). After stirring for 30 min at room temperature the reaction mixture was quenched with water (10 mL). The reaction mixture was then extracted with diethyl ether. The organic phase was washed with water, brine and dried over anhydrous sodium sulphate. Solvent was removed *in vacuo*. No further purification

was necessary. The product was obtained as pale yellow crystals, 187 mg (90%), Mp. 155-158 °C (lit. Mp. 158 °C [29]). IR$_{Vmax}$ (ATR): 3372 (O-H), 3045, 2970 (C-H), 1621, 1435 (C=C) cm^{-1}. ^{1}H NMR (400 MHz, $CDCl_3$) δ 1.75 (br. s., 1 H, OH), 5.66 (s, 2 H, CH_2), 7.42 - 7.64 (m, 4 H, 4 x ArH), 8.04 (d, *J* = 7.93 Hz, 2 H, 2 x ArH), 8.33 - 8.54 (m, 3 H, 3 x ArH). ^{13}C NMR (101 MHz, $CDCl_3$) 57.4 (CH_2), 123.9 (CH), 125.1 (CH), 126.4 (CH), 128.4 (CH), 129.1 (CH), 130.2, 131.0, 131.5 ppm. HRMS (APCI) calculated for $C_{15}H_{11}O$ [M^+-H] 207.0815: found 207.0805.

9-(Chloromethyl)anthracene (27)

To a solution of anthracen-9-ylmethanol (1.2 g, 5.77 mmol) in dry dioxane (10 mL) was added thionyl chloride (0.6 mL). The reaction mixture was then refluxed for 6 h. The reaction was allowed to come to room temperature and the reaction mixture was concentrated *in vacuo*. The remaining solids were washed with hexane, filtered and dried to afford the product as yellow crystals 1.21 g (93%), Mp. 138-140 °C (lit. Mp. 141-142.5 °C [30]). IR$_{Vmax}$ (ATR): 2970, 2884 (C-H), 1623, 1505 (C=C) cm^{-1}. ^{1}H NMR (400 MHz, $CDCl_3$) δ 5.63 (s, 2 H, CH_2), 7.46 - 7.58 (m, 2 H, 2 x ArH), 7.59 - 7.68 (m, 2 H, 2 x ArH), 8.05 (d, *J* = 7.93 Hz, 2 H, 2 x ArH), 8.34 (d, *J* = 8.55 Hz, 2 H, 2 x ArH), 8.50 (s, 1 H, H10). ^{13}C NMR (101 MHz, $CDCl_3$) 38.9 (CH_2), 123.4 (CH), 125.2 (CH), 126.9 (CH), 127.6, 129.2 (CH), 129.3 (CH), 129.9, 131.4 ppm. HRMS (APCI) calculated for $C_{15}H_{10}Cl^{35}$ [M^++H] 225.0477: found 225.0466.

2-(Anthracen-9-yl)acetonitrile (28)

To a solution of 9-(chloromethyl)anthracene (1.5 g, 6.6 mmol) in acetonitrile (160 mL) was added potassium cyanide (6.6 mmol). The reaction mixture was refluxed for 2 h, cooled and then filtered. The solvent was removed *in vacuo* and the residue was dissolved in dichloromethane. The organic layer was washed with water, brine and dried over magnesium sulphate. Solvent was removed *in vacuo* to give pure product as a yellow solid, 1.29 g (90%), Mp. 161-162 °C (lit. Mp. 159-163 °C [30]). IR$_{Vmax}$ (ATR): 3087, 2981 (C-H), 2240 (CN), 1658 (C=C), 1511, 1472 (C=C) cm^{-1}. ^{1}H NMR (400 MHz, $CDCl_3$) δ 4.54 (s, 2 H, CH_2), 7.45 - 7.54 (m, 2 H, 2 x ArH), 7.57 - 7.65 (m, 2 H, 2 x ArH), 8.02 (d, *J* = 7.93 Hz, 2 H, 2 x ArH), 8.13 (d, *J* = 9.16 Hz, 2 H, 2 x ArH), 8.47 (s, 1 H, H10). ^{13}C NMR (101 MHz, $CDCl_3$) 16.1 (CH_2), 117.7 (CN), 120.7, 122.9 (CH), 125.2 (CH), 127.2 (CH), 128.8 (CH), 129.4 (CH), 129.7, 131.3 ppm. HRMS (APCI) calculated for $C_{16}H_{12}N$ [M^++H] 218.0970: found 218.0964.

General procedure for preparation of (anthracen-9-yl)methylamines (29a – 29d)

To a solution of 9-chloromethylanthracene (225 mg, 1 mmol) in dry dichloromethane (5 mL) was added the required amine (2 mmol). The reaction mixture was stirred at room temperature for 24 h. The reaction mixture was then poured onto water and extracted into dichloromethane. The organic layer was washed with water, brine and dried over anhydrous sodium sulphate. Solvent was removed *in vacuo* and product was recrystallized from dichloromethane:hexane (1:3).

1-(Anthracen-9-ylmethyl)azepane (29a)

1-(Anthracen-9-ylmethyl)azepane was prepared from 9-(chloromethyl)anthracene (0.23 g, 1 mmol) and homopiperidine according to the general procedure above. The product was obtained as yellow crystals, 249 mg (86%), Mp. 100-101 ^{o}C (lit. Mp. 102-104 ^{o}C [31]). IR$_{Vmax}$ (ATR): 3048, 2930 (C-H), 1598, 1518 (C=C), 1195 (C-N) cm^{-1}. ^{1}H NMR (400 MHz, $CDCl_3$) δ 1.63 (s, 8 H, 4 x CH_2), 2.80 (br. s., 4 H, 2 x NCH_2), 4.56 (s, 2 H, NCH_2-anthracene), 7.41 - 7.61 (m, 4 H, 4 x ArH), 8.03 (d, *J* = 8.55 Hz, 2 H, 2 x ArH), 8.43 (s, 1 H, H10), 8.59 (d, *J* = 8.55 Hz, 2 H, 2 x ArH). ^{13}C NMR (101 MHz, $CDCl_3$) 27.2 (CH_2), 28.3 (CH_2), 53.9 (CH_2), 54.9 (CH_2), 124.7 (CH), 125.3 (CH), 125.4 (CH), 127.1 (CH), 128.8 (CH), 131.3, 131.3, 131.4 ppm. HRMS (APCI) calculated for $C_{21}H_{24}N$ [M^{+}+H] 290.1909: found 290.1899.

1-(Anthracen-9-ylmethyl)pyrrolidine (29b)

1-(Anthracen-9-ylmethyl)pyrrolidine was prepared from 9-(chloromethyl)anthracene (0.23 g, 1 mmol) and pyrrolidine according to general procedure above. The product was isolated as yellow crystals 196 mg (75%), Mp. 109-110 ^{o}C (lit. Mp. 108-110 ^{o}C [31]). IR$_{Vmax}$ (ATR): 3083, 2919 (C-H), 1572, 1490.91 (C=C), 1232 (C-N) cm^{-1}. ^{1}H NMR (400 MHz, $CDCl_3$) δ 1.77 (dt, *J* = 6.56, 3.13 Hz, 4 H, 2 x CH_2N), 2.63 - 2.75 (m, 4 H, 2 x CH_2), 4.61 (s, 2 H, NCH_2), 7.38 - 7.57 (m, 4 H, 4 x ArH), 8.02 (d, *J* = 8.55 Hz, 2 H, 2 x ArH), 8.42 (s, 1 H, H10), 8.56 (d, *J* = 9.16 Hz, 2 H, 2 x ArH). ^{13}C NMR (101 MHz, $CDCl_3$) 23.6 (C3", C4"), 51.4 (C1'), 54.3 (C2", C5"), 124.8 (CH), 125.0 (CH), 125.4 (CH), 127.1 (CH), 128.9 (CH), 130.9, 131.2, 131.4 ppm. HRMS (APCI) calculated for $C_{19}H_{20}N$ [M^{+}+H] 262.1596: found 262.1590.

Anthracen-9-yl(morpholino)methanone (29c)

Anthracen-9-yl(morpholino)methanone was prepared from 9-(chloromethyl) anthracene (0.23 g, 1 mmol) and morpholine according to general procedure above. The product was obtained as yellow crystals, 250 mg (90%), Mp. 118-122 ^{o}C (lit. Mp. 122-124 ^{o}C [31]). IR$_{Vmax}$ (ATR): 2924, 2854 (C-H), 1622, 1422 (C=C), 1110 (C-N) cm^{-1}. ^{1}H

NMR (400 MHz, $CDCl_3$) δ 2.57 - 2.71 (m, 4 H, 2 x CH_2), 3.58 - 3.75 (m, 4 H, 2 x CH_2), 4.47 (s, 2 H, C1'H_2), 7.44 - 7.60 (m, 4 H, 4 x ArH), 8.02 (d, *J* = 7.93 Hz, 2 H, 2 x ArH), 8.44 (s, 1 H, H10), 8.50 (d, *J* = 9.16 Hz, 2 H, 2 x ArH). ^{13}C NMR (101 MHz, $CDCl_3$) 53.6 (CH_2), 54.6 (CH_2), 67.1 (CH_2), 124.8 (CH), 125.0 (CH), 125.6 (CH), 127.2 (CH), 127.6 (CH), 129.0 (CH), 131.3, 131.4, 134.1 (CH) ppm. HRMS (APCI) calculated for $C_{19}H_{20}NO$ [M^++H] 278.1545: found 278.1539.

1-(Anthracen-9-ylmethyl)pyrrolidine (29d)

1-(Anthracen-9-ylmethyl)pyrrolidine was prepared from 9-(chloromethyl)anthracene (0.23 g, 1 mmol) and pyrrolidine according to the general procedure above. The product was obtained as yellow crystals, 226 mg (82%), Mp. 117-118 °C (lit. Mp. 119-121 °C [31]). IR$_{Vmax}$ (ATR): 3049, 2849 (C-H), 1599, 1449 (C=C), 1118 (C-N) cm^{-1}. ^{1}H NMR (400 MHz, $CDCl_3$). δ 1.39 - 1.58 (m, 6 H, 3 x CH_2), 2.57 (br. s., 4 H, 2 x NCH_2), 4.38 (s, 2 H, NCH_2), 7.38 - 7.58 (m, 4 H, 4 x ArH), 7.99 (d, *J* = 7.94 Hz, 2 H, 2 x ArH), 8.40 (s, 1 H, H10), 8.53 (d, *J* = 8.55 Hz, 2 H, 2 x ArH). ^{13}C NMR (101 MHz, $CDCl_3$) 24.6 (C4”), 26.2 (C3”, C5”), 54.8 (C2”, C6”), 55.1 (C1’), 124.7 (CH), 125.3 (CH), 127.1 (CH), 128.8 (CH), 130.6, 131.4, 131.4 ppm. HRMS (APCI) calculated for $C_{20}H_{22}N$ [M^++H] 276.1752: found 276.1745.

General procedure for preparation of anthracene amides via the Mukaiyama reaction (33a, 33d – 33f)

To a suspension of anthracene-9-carboxylic acid (0.22 g, 1 mmol) in anhydrous dichloromethane (10 mL) was added chloro-1-methylpyridinium iodide (0.76 g, 3 mmol). The solution turned yellow and was stirred for a further five min. To this was added the required amine (1 mmol) followed by the addition of trimethylamine (0.7 mL, 5 mmol). The reaction mixture was stirred at room temperature for 1 h and was then diluted with 10% hydrochloric acid (10 mL) followed by extraction with dichloromethane (10 mL). The organic layer was washed with 10% sodium hydroxide solution, water and brine. The solvent was removed *in vacuo* and the residue was purified by column chromatography (eluent: dichloromethane) and recrystallized from ethanol.

N,N-Diethylanthracene-9-carboxamide (33a)

N,N-Diethylanthracene-9-carboxamide was prepared from 9-anthracenecarboxylic acid (0.2 g, 1 mmol) and N,N-diethylamine according to general procedure above. The

product was obtained as a brown solid, 236 mg (85%), Mp. 188-190 °C (lit. Mp. 189-192 °C [32]). IR$_{Vmax}$ (ATR): 3049, 2970, 2931 (C-H), 1623, 1486 (C=C), 1257 (C-N) cm^{-1}. ^1H NMR (400 MHz, $CDCl_3$) δ 0.85 (t, *J* = 7.02 Hz, 3 H, CH_3), 1.52 (t, *J* = 7.02 Hz, 3 H, CH_3), 2.88 - 3.11 (m, 2 H, CH_2), 3.77 - 4.01 (m, 2 H, CH_2), 7.36 - 7.61 (m, 4 H, 4 x ArH), 7.93 (d, *J* = 8.55 Hz, 2 H, 2 x ArH), 8.01 (d, *J* = 7.94 Hz, 2 H, 2 x ArH), 8.45 (s, 1 H, H10). ^{13}C NMR (101 MHz, $CDCl_3$) 13.1 (CH_3), 14.1 (CH_3), 39.1 (CH_2), 43.1 (CH_2), 124.9 (CH), 125.4 (CH), 126.5 (CH), 127.3 (CH), 127.5, 128.5 (CH), 131.2 (s), 131.5 (s), 169.6 (C=O) ppm. HRMS (APCI) calculated for $C_{19}H_{20}NO$ [M^++H] 278.1539: found 278.1528.

N,N-Dimethylanthracene-9-carboxamide (33b)

N,N-Dimethylanthracene-9-carboxamide was prepared from 9-anthracenecarboxylic acid (0.2 g, 1 mmol) and dimethylamine according to general procedure above. The product was obtained as a brown solid, 200 mg (80%), Mp. 150-152 °C (lit. Mp. 151.3-153.5 °C [33]). IR$_{Vmax}$ (ATR): 3058, 2853 (C-H), 1629, 1560 (C=C), 1262 (C-N) cm^{-1}. ^1H NMR (400 MHz, $CDCl_3$) δ 2.71 (s, 3 H, CH_3), 3.43 (s, 3 H, CH_3), 7.44 - 7.57 (m, 4 H, 4 x ArH), 7.88 (d, *J* = 8.55 Hz, 2 H, 2 x ArH), 8.03 (d, *J* = 7.93 Hz, 2 H, 2 x ArH), 8.47 (s, 1 H, H10). ^{13}C NMR (101 MHz, $CDCl_3$) 34.7 (CH_3), 38.1 (CH_3), 124.9 (CH), 125.5 (CH), 126.7 (CH), 127.5, 127.5 (CH), 128.6 (CH), 131.1, 170.4 (C1') ppm. HRMS (APCI) calculated for $C_{17}H_{16}NO$ [M^++H] 250.1232: found 250.1223.

Anthracen-9-yl(pyrrolidin-1-yl)methanone (33d)

Anthracen-9-yl(pyrrolidin-1-yl)methanone was prepared from 9-anthracenecarboxylic acid (0.2 g, 1 mmol) and pyrrolidine according to general procedure above. The product was obtained as yellow crystals, 207 mg (75%), Mp. 149-151 °C (lit. Mp. 150-152 °C [33]). IR$_{Vmax}$ (ATR): 3048, 2969 (C-H), 1616, 1578 (C=C), 1224 (C-N) cm^{-1}. ^1H NMR (400 MHz, $CDCl_3$) δ 1.80 (quin, *J* = 6.87 Hz, 2 H, CH_2), 2.05 (quin, *J* = 6.87 Hz, 2 H, CH_2), 2.93 (t, *J* = 7.02 Hz, 2 H, CH_2), 3.98 (t, *J* = 7.02 Hz, 2 H, CH_2), 7.43 - 7.55 (m, 4 H, 4 x ArH), 7.93 (d, *J* = 8.55 Hz, 2 H, 2 x ArH), 7.98 - 8.06 (m, 2 H, 2 x ArH), 8.46 (s, 1 H, H10). ^{13}C NMR (101 MHz, $CDCl_3$) 24.7 (C3", C4"), 25.9 (C3", C4"), 45.6 (C5", C2"), 47.6 (C5", C2"), 124.8 (CH), 125.4 (CH), 126.7 (CH), 127.2, 127.5 (CH), 128.6 (CH), 131.23, 132.3, 168.7 (C1') ppm. HRMS (APCI) calculated for $C_{19}H_{18}NO$ [M^++H] 276.1388: found 276.1380.

Anthracen-9-yl(morpholino)methanone (33e)

Anthracen-9-yl(morpholino)methanone was prepared from 9-anthracenecarboxylic acid (0.2 g, 1 mmol) and morpholine according to general procedure above. The product was

obtained as yellow crystals, 256 mg (88%), Mp. 160-163 °C (lit. Mp. 161.5-163.5 °C [33]). IR$_{Vmax}$ (ATR): 3084, 2970 (C-H), 1610, 1487 (C=C), 1234 (C-N) cm^{-1}. ^{1}H NMR (400 MHz, $CDCl_3$) δ 3.01 - 3.09 (m, 2 H, CH_2), 3.41 - 3.48 (m, 2 H, CH_2), 3.88 - 4.00 (m, 2 H, CH_2), 4.09 - 4.19 (m, 2 H, CH_2), 7.43 - 7.59 (m, 4 H, 4 x ArH), 7.94 (d, *J* = 8.55 Hz, 2 H, 2 x ArH), 8.03 (d, *J* = 8.55 Hz, 2 H, 2 x ArH), 8.48 (s, 1 H, H10). ^{13}C NMR (101 MHz, $CDCl_3$) 42.0 (CH_2), 47.0 (CH_2), 67.0 (CH_2), 67.1 (CH_2), 124.6 (CH), 125.6 (CH), 126.9 (CH), 127.6, 127.9 (CH), 128.7 (CH), 129.9, 131.1, 168.8 (C1') ppm. HRMS (APCI) calculated for $C_{19}H_{18}NO$ [M^{+}+H] 292.1338: found 292.1328.

Anthracen-9-yl(piperidin-1-yl)methanone (33f)

Anthracen-9-yl(piperidin-1-yl)methanone was prepared from 9-anthracenecarboxylic acid (0.2 g, 1 mmol) and piperidine according to general procedure above. The product was obtained as yellow crystals, 261 mg (90%), Mp. 181-183 °C (lit. Mp. 183.2-185 °C [33]). IR$_{Vmax}$ (ATR): 3051, 2992 (C-H), 1753 (C=O), 1655, 1458 (C=C), 1222 (C-N) cm^{-1}. ^{1}H NMR (400 MHz, $CDCl_3$) δ 1.27 - 1.40 (m, 2 H, CH_2), 1.61 - 1.75 (m, 2 H, CH_2), 1.85 (dt, *J* = 11.29, 5.95 Hz, 2 H, CH_2), 2.96 - 3.11 (m, 2 H, CH_2), 3.99 - 4.15 (m, 2 H, CH_2), 7.38 - 7.57 (m, 4 H, 4 x ArH), 7.95 (d, *J* = 7.93 Hz, 2 H, 2 x ArH), 7.98 - 8.13 (m, 2 H, 2 x ArH), 8.46 (s, 1 H, H10). ^{13}C NMR (101 MHz, $CDCl_3$) 24.5 (C4"), 26.0 (C3", C5"), 26.7 (C3", C5"), 42.5 (C2", C6"), 47.9 (C2", C6"), 125.0 (CH), 125.5 (CH), 126.5 (CH), 127.3 (CH), 127.5, 128.6 (CH), 131.2, 131.2, 168.5 (C1') ppm. HRMS (APCI) calculated for $C_{20}H_{20}NO$ [M^{+}+H] 290.1536: found 290.1545.

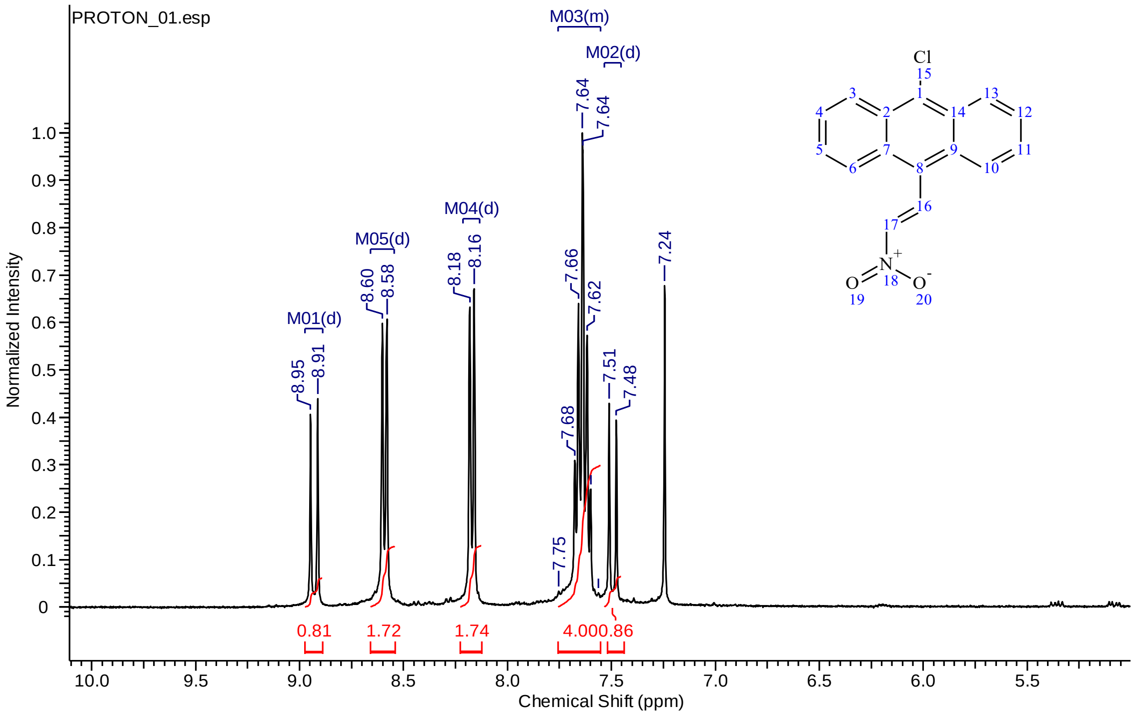

Figure S1: ^{1}H NMR spectrum of (*E*)-9-Chloro-10-(2-nitrovinyl)anthracene (19d)

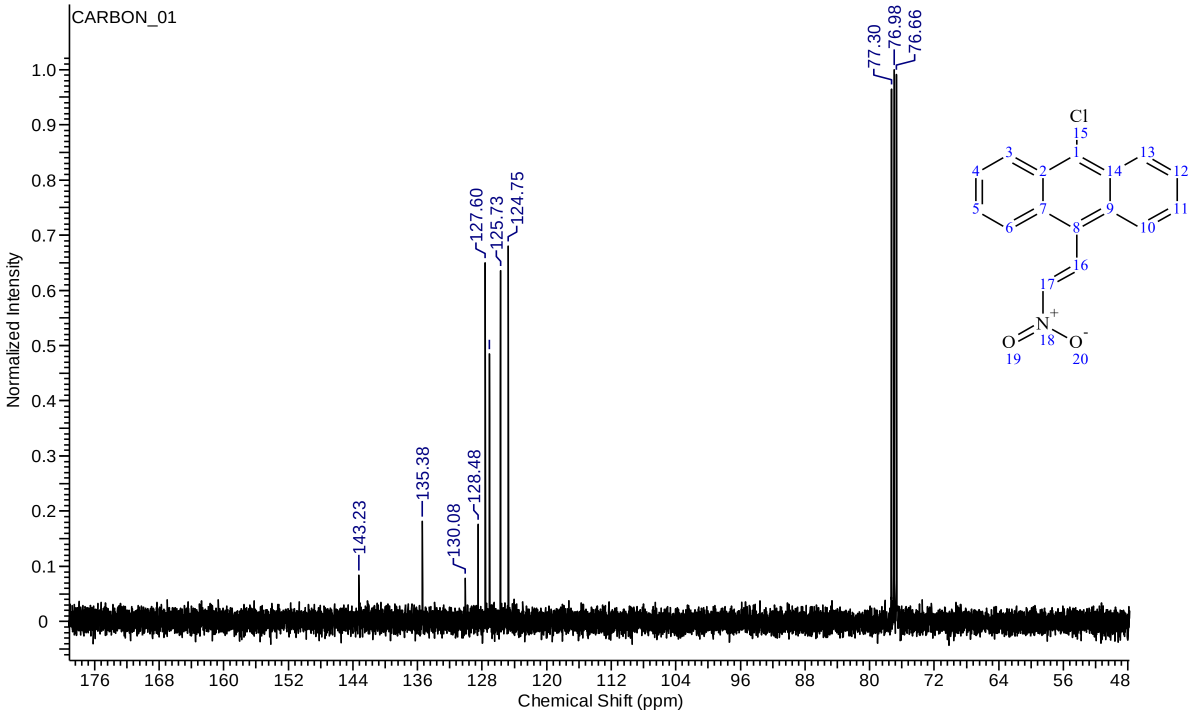

Figure S2: ^{13}C NMR spectrum of (*E*)-9-Chloro-10-(2-nitrovinyl)anthracene (19d)

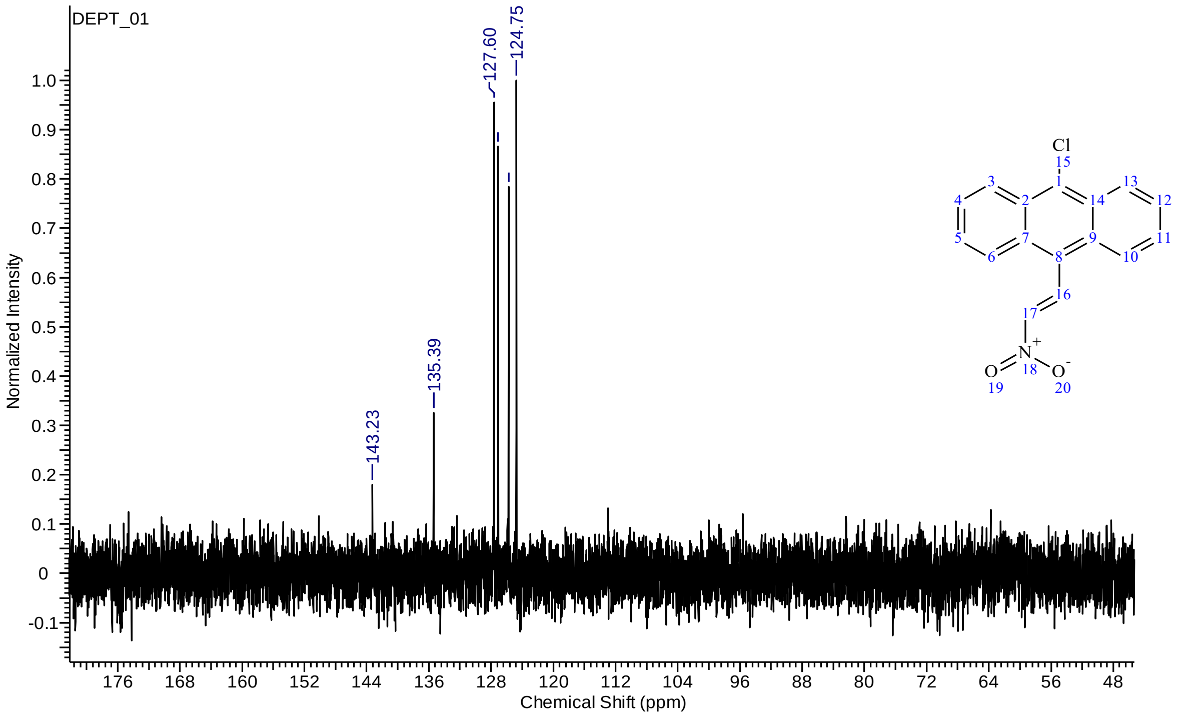

Figure S3: DEPT-90 spectrum of (*E*)-9-Chloro-10-(2-nitrovinyl)anthracene (19d)

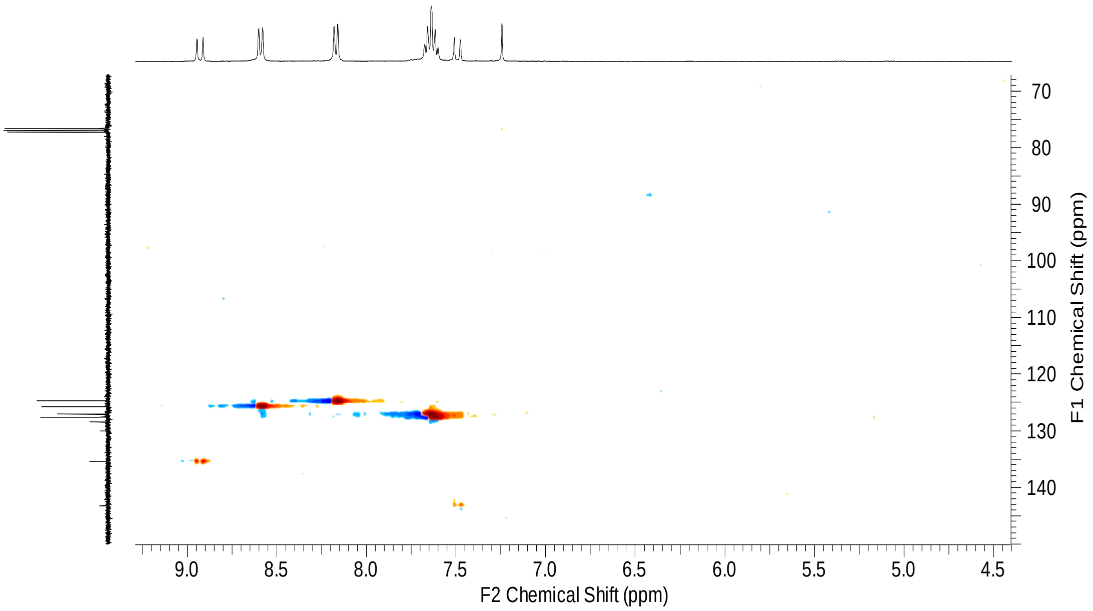

Figure S4: HSQC spectrum of (*E*)-9-Chloro-10-(2-nitrovinyl)anthracene (19d)

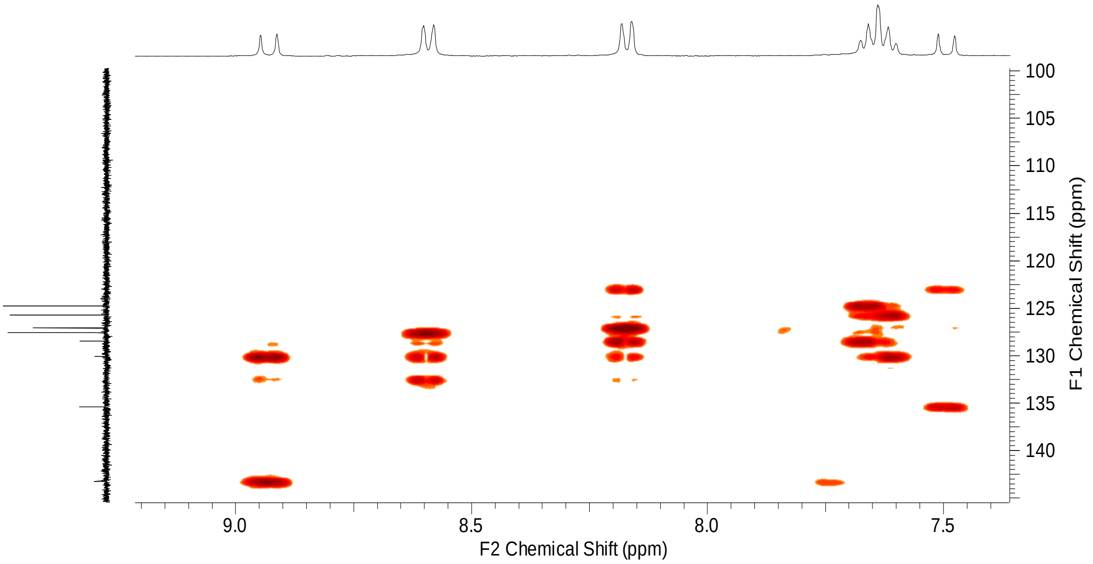

Figure S5: HMBC spectrum of (*E*)-9-Chloro-10-(2-nitrovinyl)anthracene (19d)

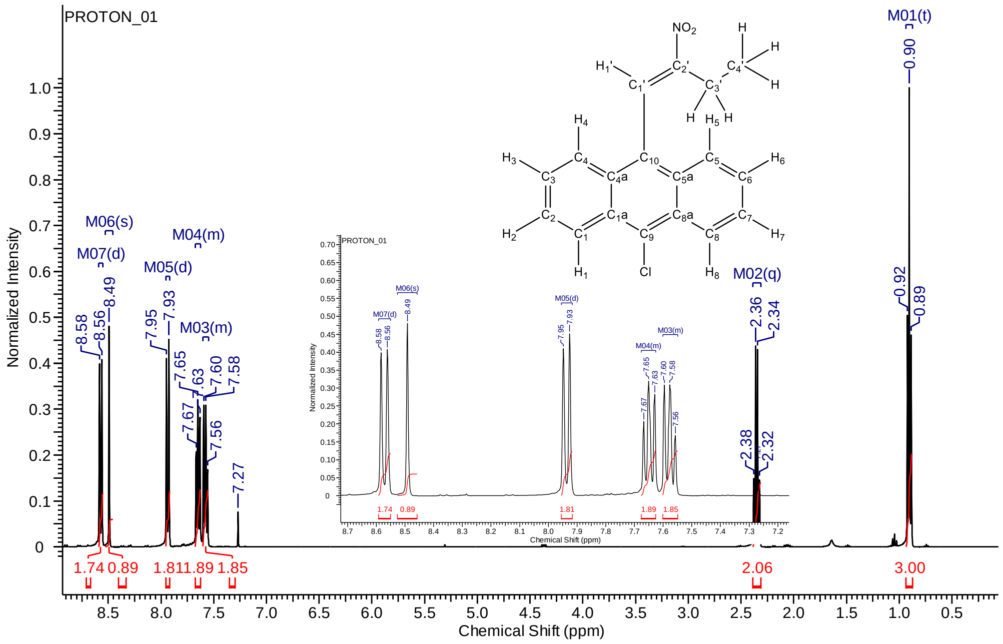

Figure S6: ^{1}H NMR spectrum of (*E*)-9-chloro-10-(2-nitrobut-1-en-1-yl) anthracene (19f)

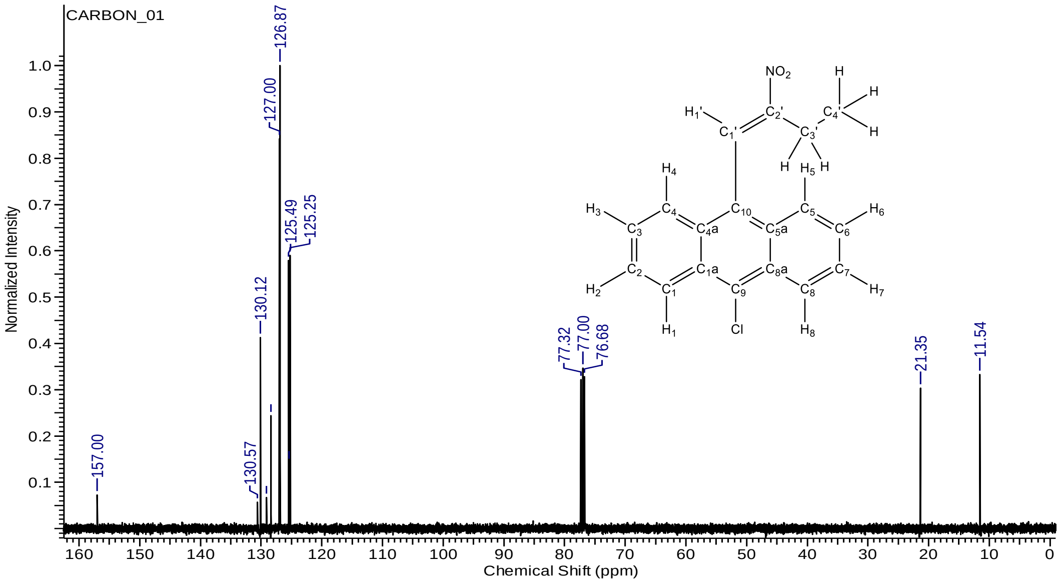

Figure S7: ^{13}C NMR spectrum of (*E*)-9-chloro-10-(2-nitrobut-1-en-1-yl) anthracene (19f)

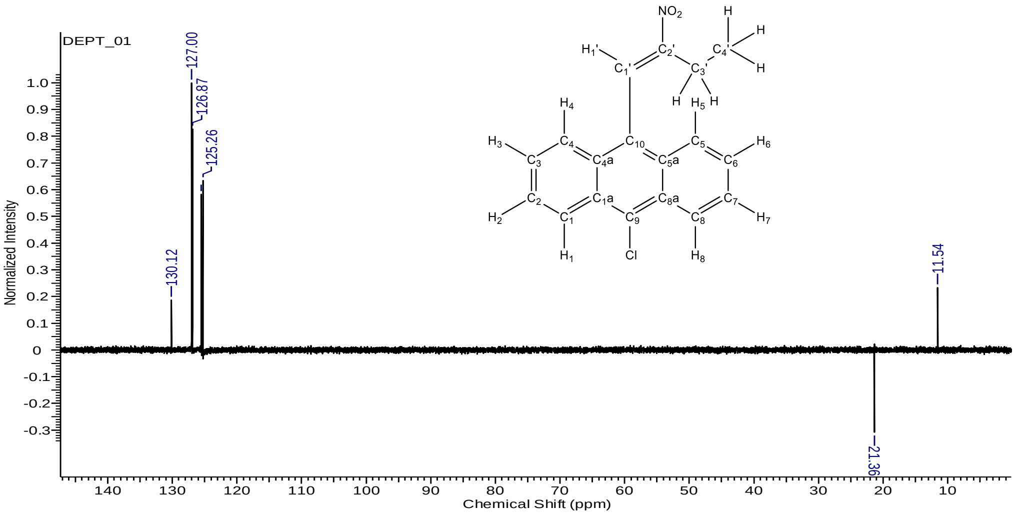

Figure S8: DEPT 135 NMR spectrum of (*E*)-9-chloro-10-(2-nitrobut-1-en-1-yl) anthracene (19f)

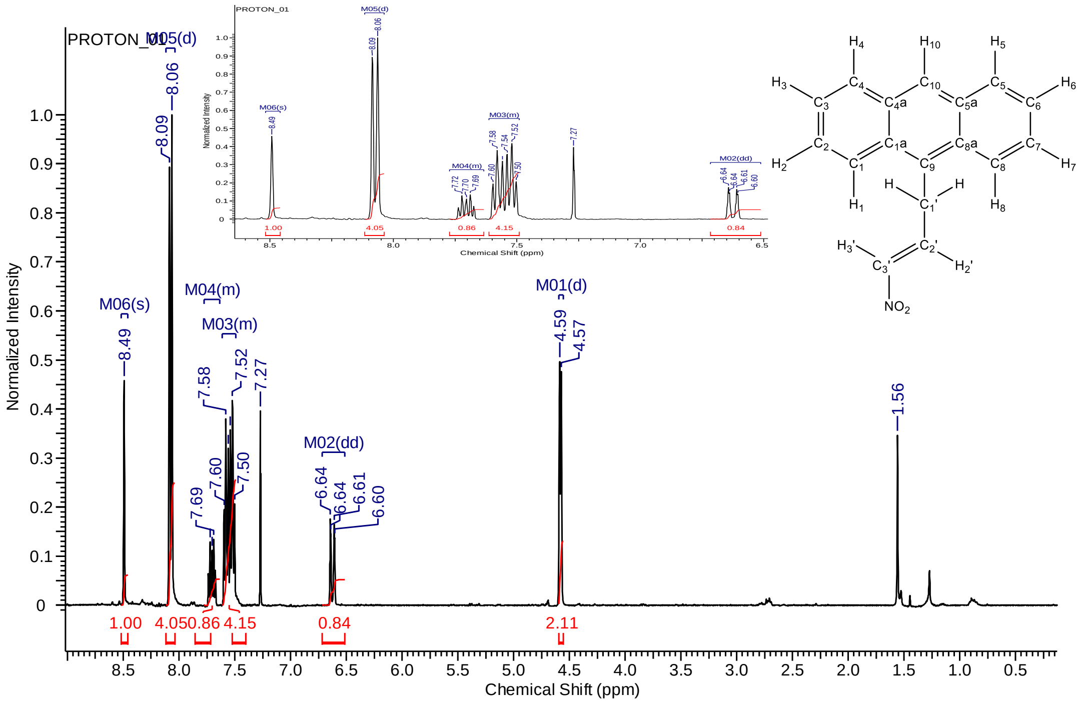

Figure S9: ^{1}H NMR spectrum of (*E*)-9-(3-nitroallyl)anthracene (20a)

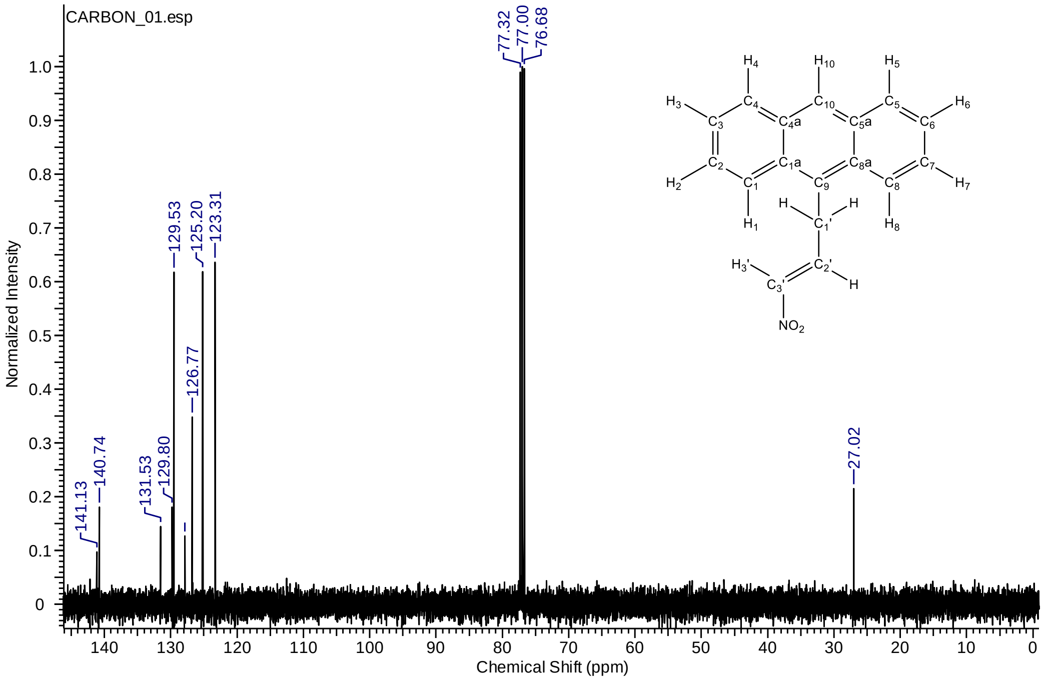

Figure S10: ^{13}C NMR spectrum of (*E*)-9-(3-nitroallyl)anthracene (20a)

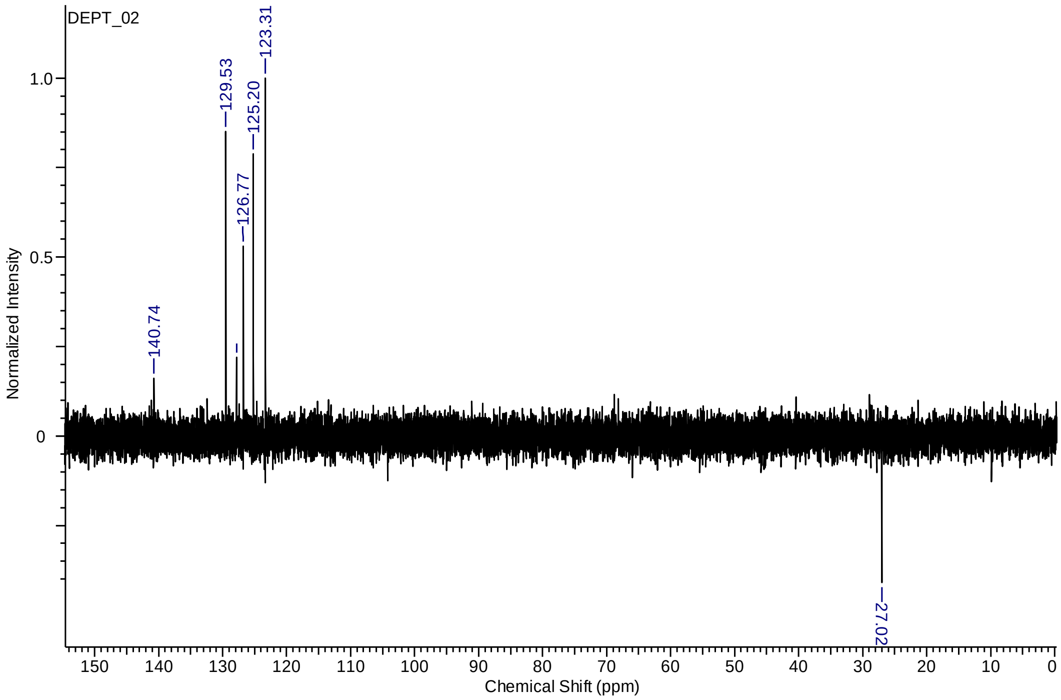

Figure S11: DEPT 135 NMR spectrum of (*E*)-9-(3-nitroallyl)anthracene (20a)

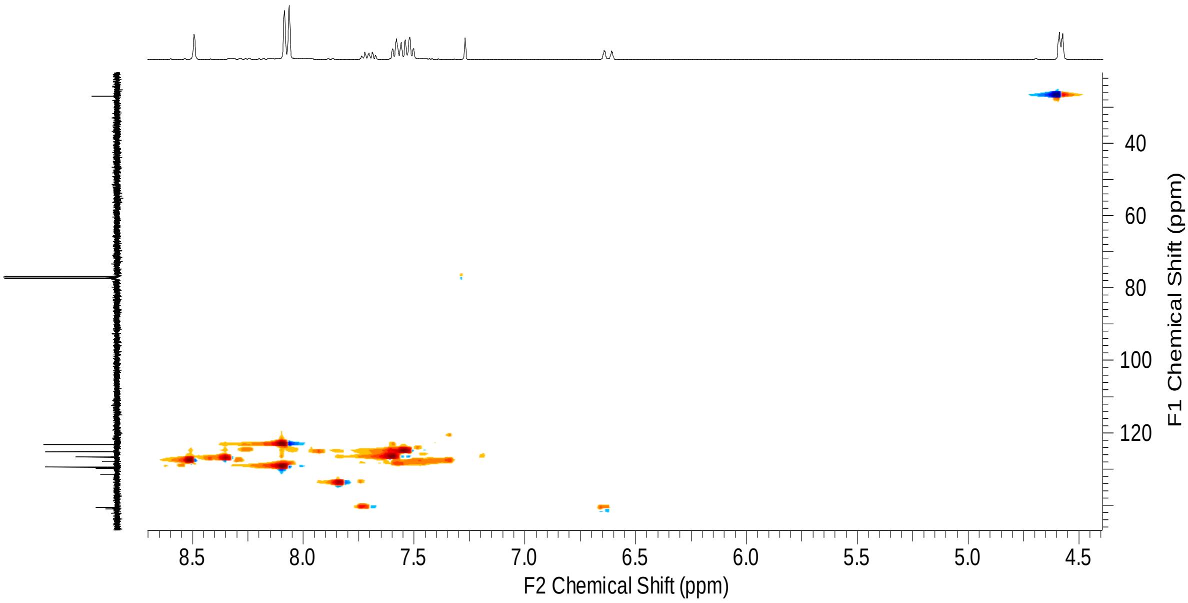

Figure S12: C-H COSY spectrum of (*E*)-9-(3-nitroallyl)anthracene (20a)

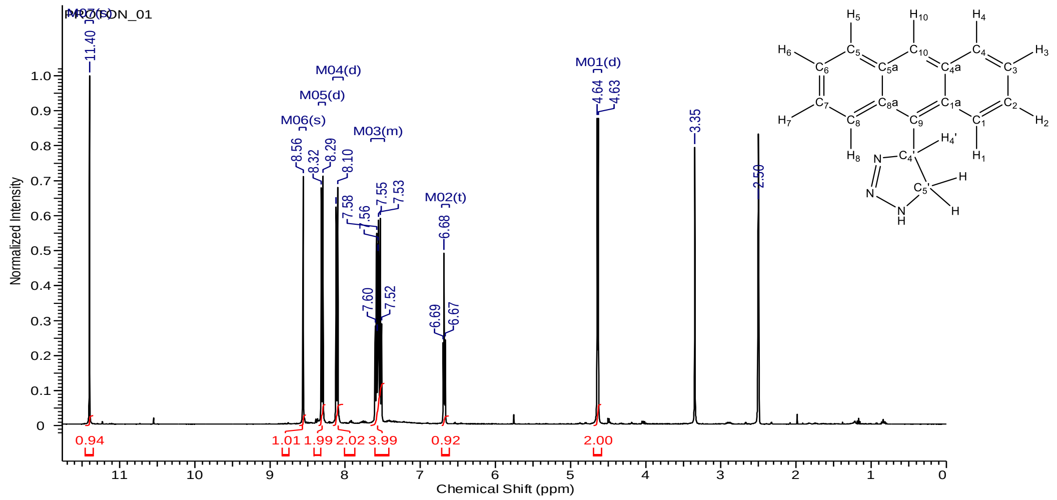

Figure S132: ^{1}H NMR spectrum of 4-(anthracen-9-yl)-4,5-dihydro-1*H*-1,2,3-triazole (31)

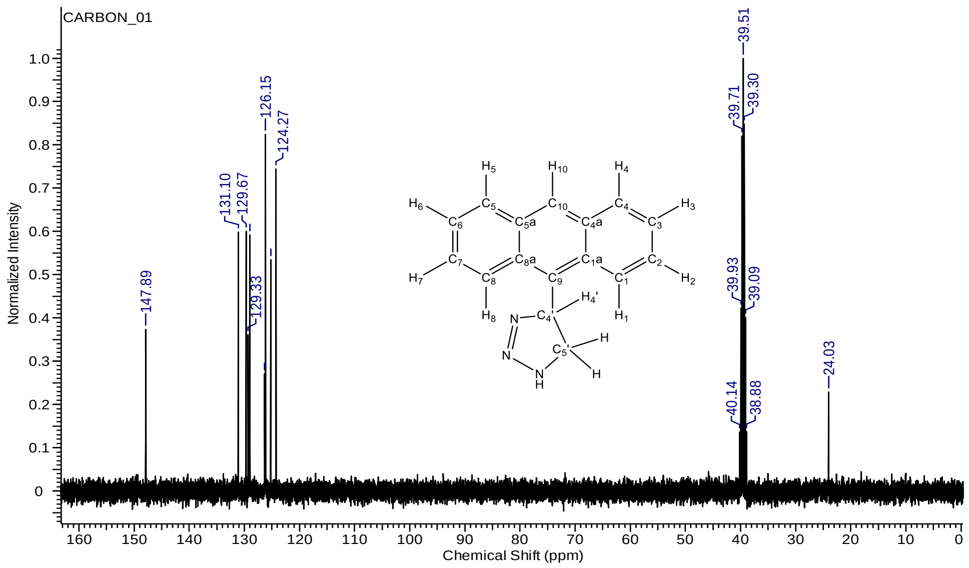

Figure S14: ^{13}C NMR spectrum of 4-(anthracen-9-yl)-4,5-dihydro-1*H*-1,2,3-triazole (31)

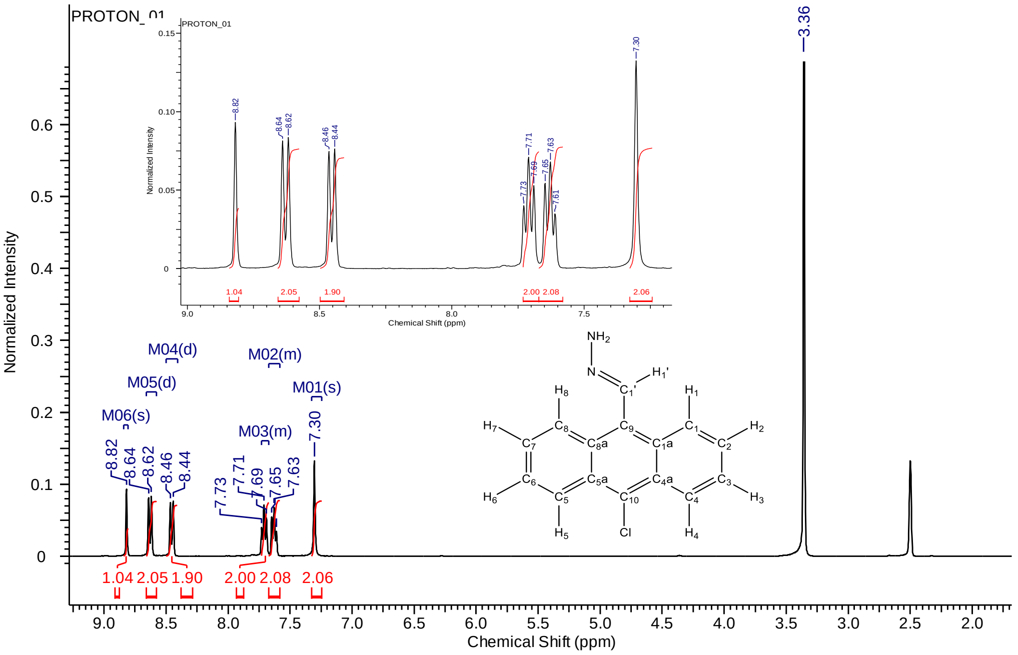

Figure S15: ^{1}H NMR spectrum of (*E*)-((10-chloroanthracen-9-yl)methylene) hydrazine (32b)

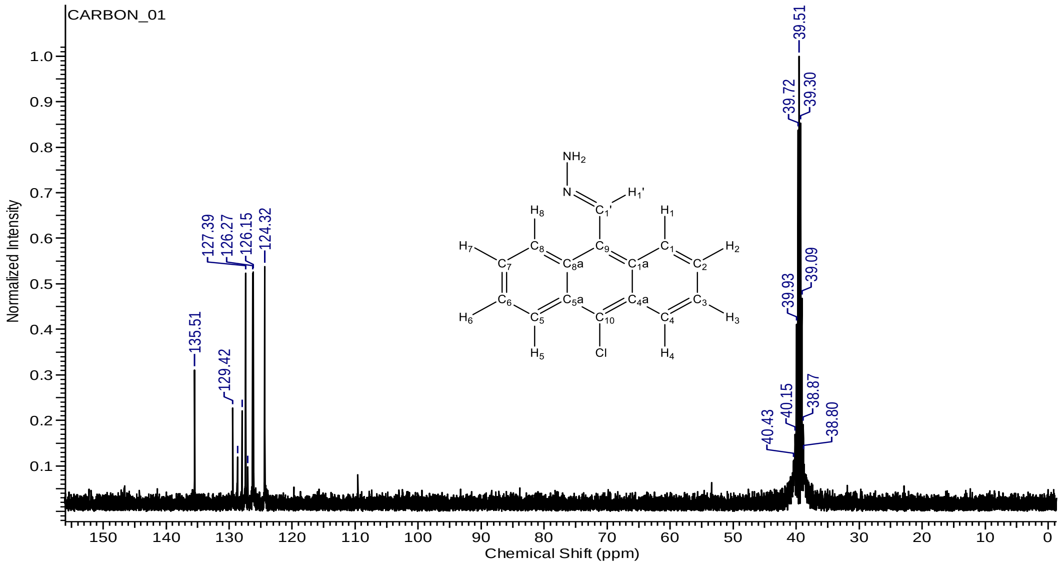

Figure S16: ^{13}C NMR spectrum of (E)-((10-chloroanthracen-9-yl)methylene) hydrazine (32b)

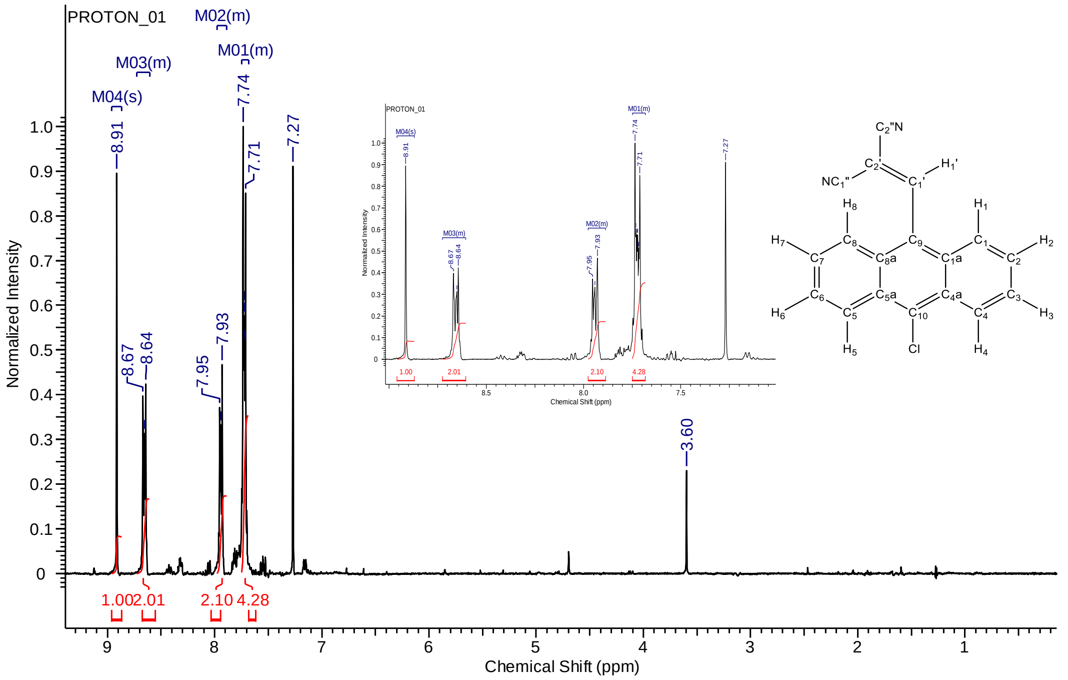

Figure S17: ^{1}H NMR spectrum of 2-((10-chloroanthracen-9-yl)methylene) malononitrile (23b)

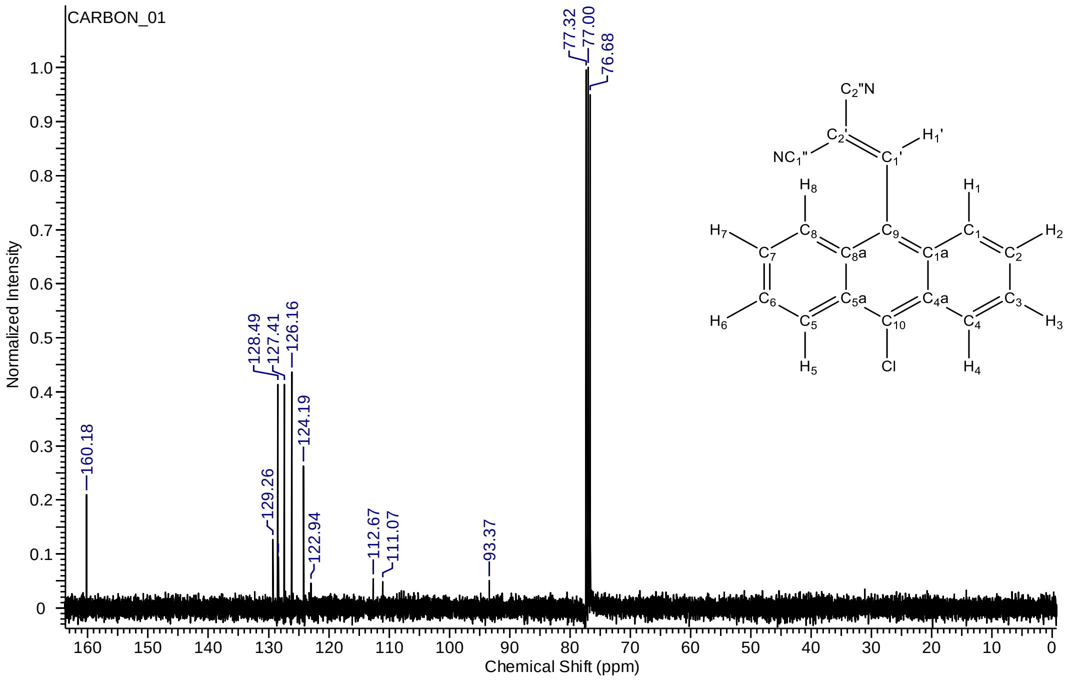

Figure S18: ^{13}C NMR spectrum of 2-((10-chloroanthracen-9-yl)methylene) malononitrile (23b)

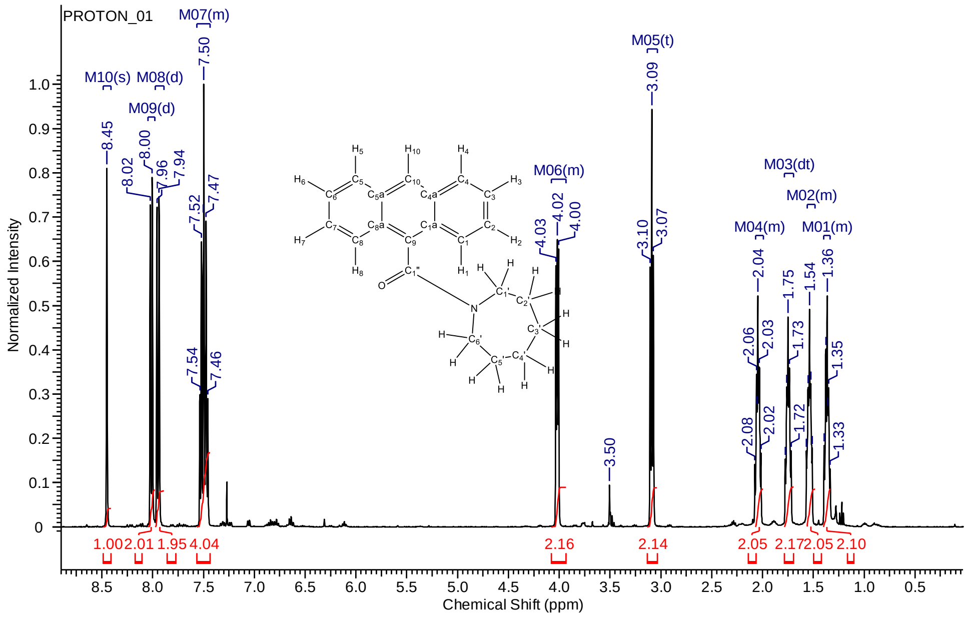

Figure S19: ^{1}H NMR spectrum of anthracen-9-yl(azepan-1-yl)methanone (33c)

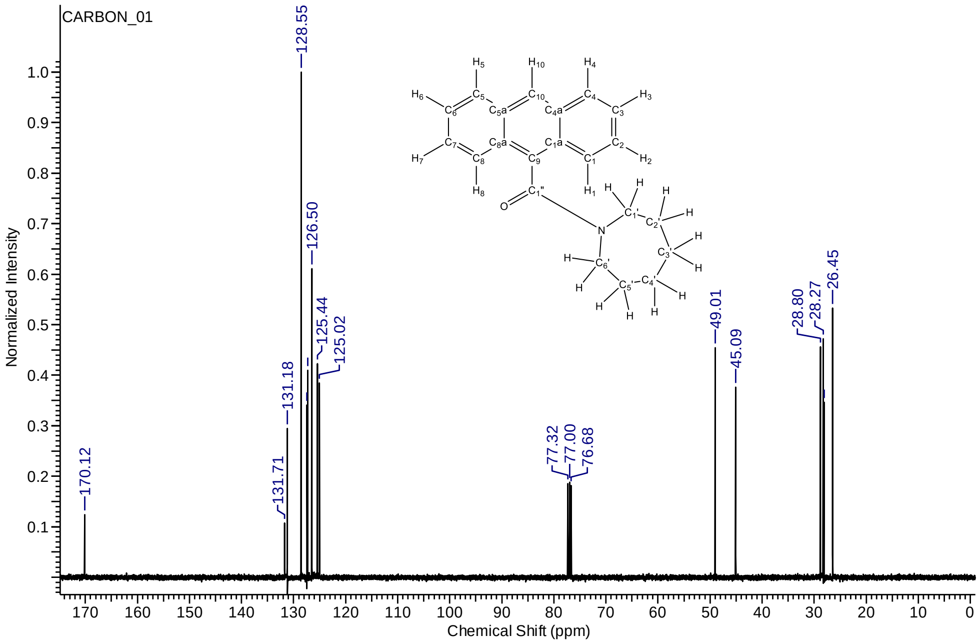

Figure S20: ^{13}C NMR spectrum of anthracen-9-yl(azepan-1-yl)methanone (33c)

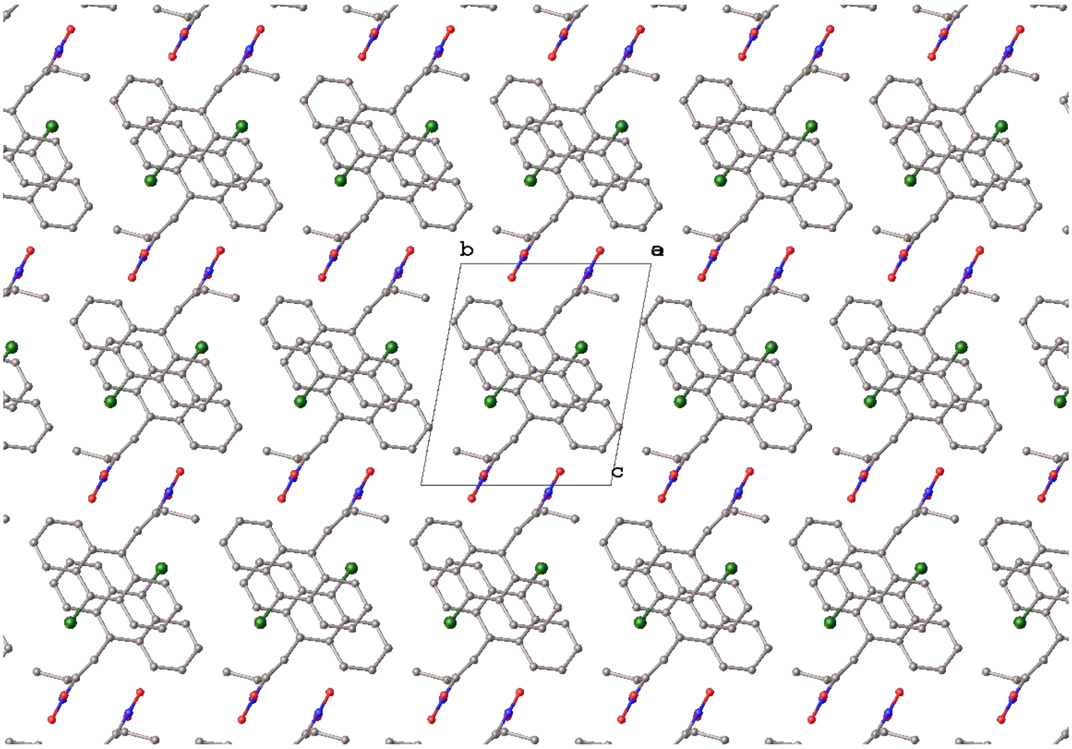

Figure S21. Schematic packing diagram of 19f viewed normal to the a-axis, with hydrogen atoms omitted for clarity

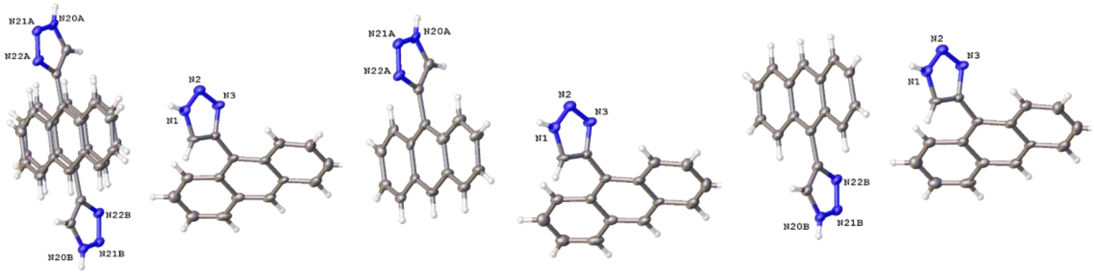

A B C

Figure S22. Molecular structure of **30a** with atomic displacement shown at 50% probability with (A) the complete disordered molecule with both moieties at 50% occupancy and (B) one unique disordered moiety only (50% occupancy) and (C) the remaining disordered moiety (50% occupancy). Only heteroatoms labelled for clarity.

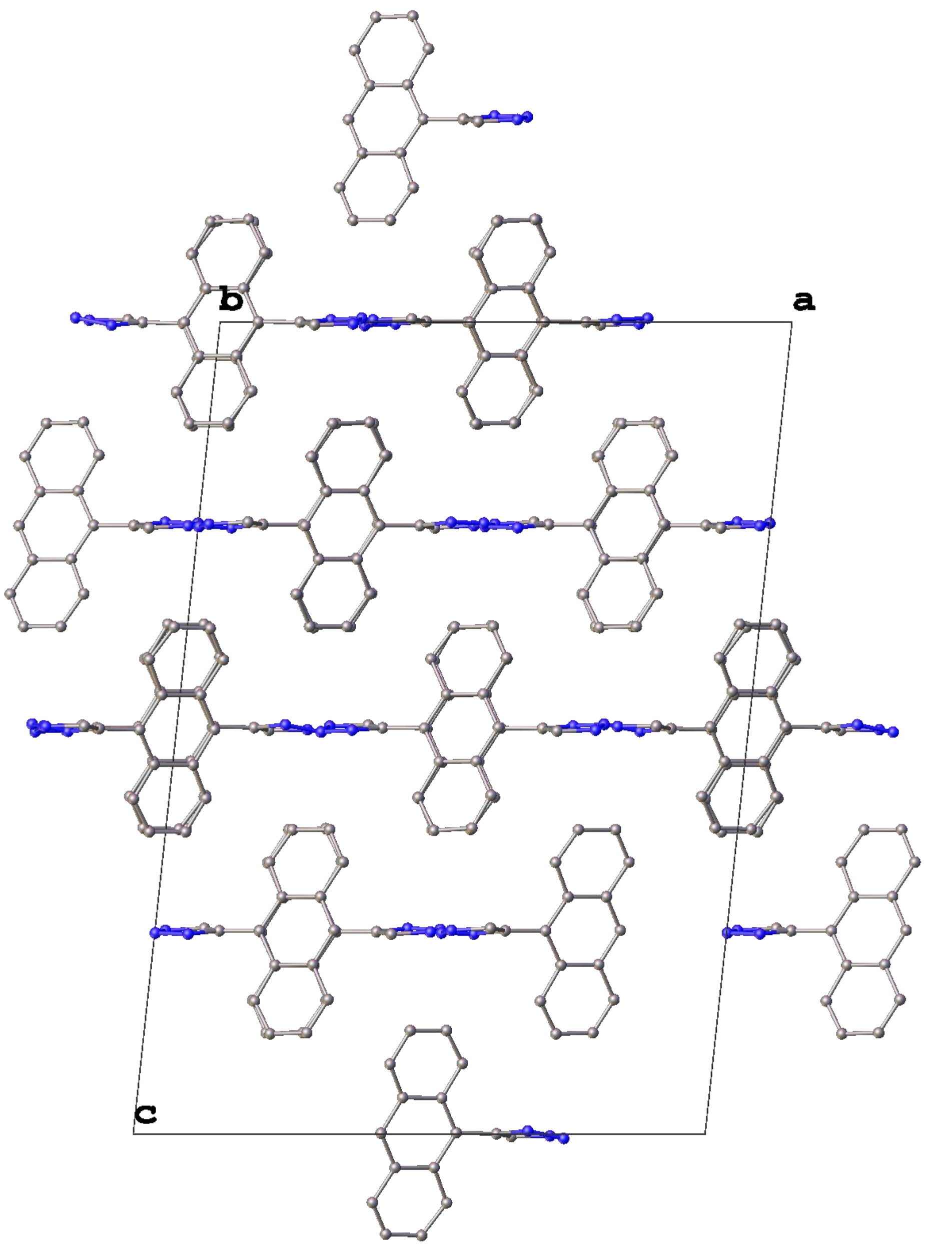

Figure S23. Schematic packing diagram of one disordered moiety of 30a viewed normal to the b-axis, with hydrogen atoms omitted for clarity

Table S1. Tier-1 Profiling Screen of selected nitrovinylanthracenes and related compounds [a]

Compound Structure	Compound Number	ADMET Solubility[b]	ADMET Solubility Level[c]	ADMET BBB[d]	ADMET BBB Level[e]	ADMET CYP2D6 Prediction[f]	ADMET Hepatotoxic Prediction[g]
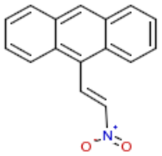	**19a**	-5.4280	2	0.28900	1	false	true
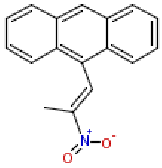	**19b**	-5.9720	2	0.44800	1	false	true
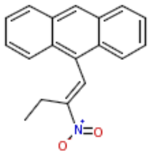	**19c**	-6.3760	1	0.61000	1	false	true
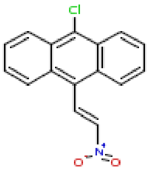	**19d**	-6.0270	1	0.49500	1	false	true
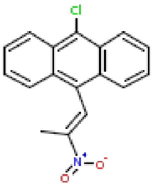	**19e**	-6.5680	1	0.65300	1	false	true
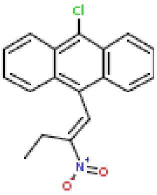	**19f**	-6.9680	1	0.81500	0	false	true
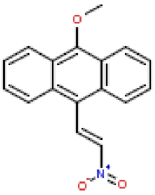	**19g**	-5.3960	2	0.14300	1	false	true

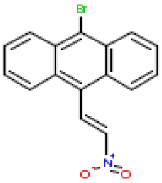	**19h**	-6.1600	1	0.52100	1	false	true
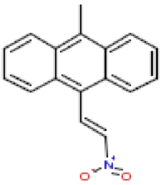	**19i**	-5.9630	2	0.44000	1	false	true
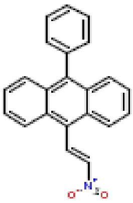	**19j**	-7.0840	1	0.75900	0	false	true
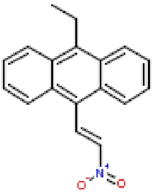	**19l**	-6.3200	1	0.58100	1	false	true
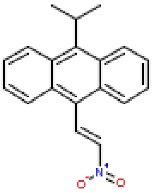	**19m**	-6.6220	1	0.65800	1	false	true
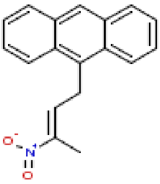	**20b**	-6.3410	1	0.58900	1	false	true
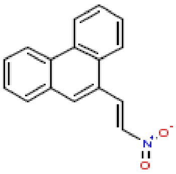	**21**	-5.4430	2	0.28900	1	false	true
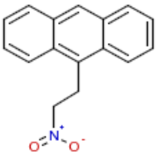	**22**	-5.7050	2	0.40900	1	false	true

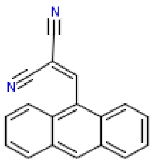	**23a**	-5.4750	2	0.30500	1	false	true
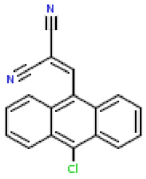	**23b**	-6.0700	1	0.51000	1	false	true
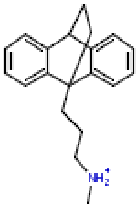	**Maprotiline**	-3.8530	3	0.73800	0	true	true

[a]Calculated using Pipeline Pilot Professional (v8.5.0.200) BIOVIA, Dassault Systèmes

[b]ADMET Solubility: Log of the water solubility at 25 °C (LogSw)(mol/L)

[c]ADMET Solubility Level: Ranking of the solubility values into the following classes: 0: Extremely Low; 1: Very Low; 2: Low; 3: Good; 4: Optimal; 5: Very Soluble

[d]ADMET BBB: Predicts the blood brain barrier penetration of a molecule, defined as the ratio of the concentrations of solute (compound) on the both sides of the membrane after oral administration.

[e]ADMET Blood Brain Barrier Absorption (BBB) Level: Ranking of LogBBB values into one of the following levels: 0: Very High; 1: High; 2: Medium; 3: Low; 4: Undefined (molecule is outside the confidence area of the regression model used to calculate LogBB)

[f]CYP2D6 inhibitor prediction

[g]Human hepatoxicity prediction

Table S2: ADMET and Lipinski properties for selected nitrovinylanthracenes and related compounds

Structure	Cpd No.	ADMET Absorption Level[b]	ADMET PPB Prediction[c]	ALogP[d]	Molecular Weight	HBA	HBD	Rot Bonds	Volume	Polar Surface Area
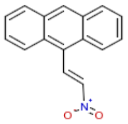	**19a**	0	true	3.6260	249.26	2	0	2	157.77	45.820
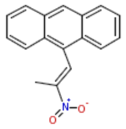	**19b**	0	true	4.1400	263.29	2	0	2	172.87	45.820
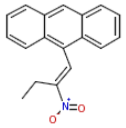	**19c**	0	true	4.6640	277.32	2	0	3	184.19	45.820
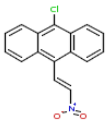	**19d**	0	true	4.2910	283.71	2	0	2	172.87	45.820
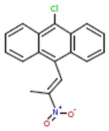	**19e**	0	true	4.8040	297.74	2	0	2	191.39	45.820
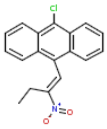	**19f**	0	true	5.3280	311.76	2	0	3	206.14	45.820
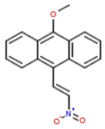	**19g**	0	true	3.6100	279.29	3	0	3	179.73	55.050
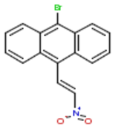	**19h**	0	true	4.3750	328.16	2	0	2	183.50	45.820
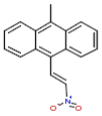	**19i**	0	true	4.1130	263.29	2	0	2	172.18	45.820
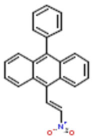	**19j**	0	true	5.1450	325.36	2	0	3	205.45	45.820

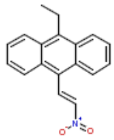	**19l**	0	true	4.5690	277.32	2	0	3	182.81	45.820
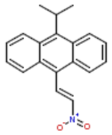	**19m**	0	true	4.8210	291.34	2	0	3	197.91	45.820
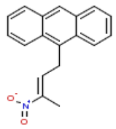	**20b**	0	true	4.5960	277.32	2	0	3	185.90	45.820
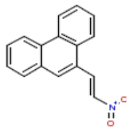	**21**	0	true	3.6260	249.26	2	0	2	156.75	45.820
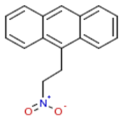	**22**	0	true	4.0120	251.28	2	0	3	160.52	45.820
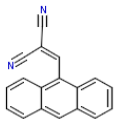	**23a**	0	true	3.8320	254.29	2	0	1	160.86	47.580
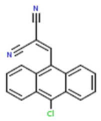	**23b**	0	true	4.4970	288.73	2	0	1	179.73	47.580
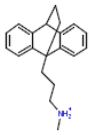	**Maprotiline**	0	true	2.8850	278.41	0	1	4	203.05	16.610

[a]Calculated using Pipeline Pilot Professional (v8.5.0.200) BIOVIA, Dassault Systèmes

[b]ADMET Calculates ADMET Passive Intestinal Absorption properties. Accelrys passive intestinal absorption model. Absorption Level: Ranking of the molecule into one of the following levels: 0: Good; 1: Moderate; 2: Poor; 3: Very Poor

[c]ADMET Plasma Protein Binding (PPB) Prediction: If true, the compound is predicted to be a binder (>=90%). Otherwise, it is predicted to be a weak or nonbinder(<90%).

[d] ChemBioDraw Ultra 13.0.2.3020

Table S3: Preliminary cell viability data for selected compound series **19a-m, 20-25, 28, 30** and **31** in MUTU-1 Burkitt lymphoma cell line (24 h)[a]

Compound	Concentration (μM)	% Viable Cells Remaining	Error
**19a**	1	82.49	2.95
**19a**	10	5.91	1.12
**19b**	1	99.83	2.64
**19b**	10	88.2	4.08
**19c**	1	88.6	2.86
**19c**	10	78.04	1.67
**19d**	1	70.8	2.5
**19d**	10	7.6	0.98
**19e**	1	104.6	2.84
**19e**	10	98.56	5.26
**19f**	1	96.57	4.64
**19f**	10	97.52	4.05
**19g**	1	90.07	4.1
**19g**	10	1.85	0.29
**19h**	1	51.17	3.2
**19h**	10	3.36	0.35
**19i**	1	94.49	3.12
**19i**	10	1.95	0.25
**19j**	1	68.52	4.54
**19j**	10	1.29	0.24
**19k**	1	70.98	4.08
**19k**	10	-0.18	0.19
**19l**	1	72.73	4.36
**19l**	10	0.5	0.21
**19m**	1	78.4	2.95
**19m**	10	0.21	0.18
**20a**	1	106.5	4.99
**20a**	10	40.37	7.07
**20b**	1	105.0	5.89
**20b**	10	14.55	5.36
**21**	1	73.62	7.12
**21**	10	1.68	0.42
**22**	1	104.9	4.92
**22**	10	100.6	2.89
**23a**	1	91.86	2.18
**23a**	10	65.29	2.44
**23b**	1	92.59	3.0
**23b**	10	16.78	1.69
**23c**	1	88.09	3.15
**23c**	10	86.91	3.07
**24**	1	102.3	2.77
**24**	10	101.4	4.11
**25**	1	107.2	4.49
**25**	10	103.2	5.16
**28**	1	95.24	2.16
**28**	10	88.8	2.68

**30a**	1	94.89	4.75
**30a**	10	94.5	3.24
**30b**	1	107.8	5.59
**30b**	10	107.3	2.06
**31**	1	96.01	3.95
**31**	10	103.1	2.8
**Maprotiline**	1	93.13	2.87
**Maprotiline**	10	72.96	3.04
**Taxol**	1	32.0	
**Taxol**	10	7.0	

[a] Cell proliferation of MUTU-1 and DG-75 cells was determined with an alamarBlue assay (seeding density 1–5 ⇥ 10^4 cells/mL per well for 96-well plates). Compound concentrations of either 1 μM or 10 μM for 24 h (MUTU-1) or 48 h (DG-75) were used to treat the cells (in triplicate) with control wells containing vehicle ethanol (1% *v*/*v*). The mean value for three independent experiments performed in triplicate is shown.

Table S4: Preliminary cell viability data for selected compound series **19a-m, 20-25, 28, 30** and **31** in DG-75 Burkitt lymphoma cell line (48 h)[a]

Compound	Concentration (μM)	% Viable Cells Remaining	Error
**19a**	1	94.63	3.78
**19a**	10	14.04	1.99
**19b**	1	102.4	2.88
**19b**	10	89.27	7.08
**19c**	1	83.78	4.57
**19c**	10	92.27	5.32
**19d**	1	75.06	1.95
**19d**	10	57.07	2.25
**19e**	1	82.54	6.27
**19e**	10	99.57	3.94
**19f**	1	105.1	5.61
**19f**	10	78.0	5.3
**19g**	1	89.21	4.54
**19g**	10	13.83	1.5
**19h**	1	84.77	3.61
**19h**	10	70.0	8.38
**19i**	1	96.68	3.19
**19i**	10	19.5	3.85
**19j**	1	83.54	3.3
**19j**	10	1.5	0.55
**19k**	1	88.65	2.92
**19k**	10	0.15	0.4
**19l**	1	104.2	6.04
**19l**	10	4.04	0.9
**19m**	1	85.23	2.92
**19m**	10	28.12	10.88
**20a**	1	99.92	3.88
**20a**	10	79.71	10.32
**20b**	1	95.41	5.04
**20b**	10	97.63	3.79
**21**	1	99.48	2.71
**21**	10	62.2	5.97
**22**	1	86.65	4.99
**22**	10	99.08	3.86
**23a**	1	103.9	3.18
**23a**	10	98.14	3.13
**23b**	1	85.59	4.03
**23b**	10	52.84	2.84
**23c**	1	84.23	2.77
**23c**	10	81.98	3.11
**24**	1	96.91	4.33
**24**	10	100.4	2.33
**25**	1	96.3	5.06
**25**	10	100.5	3.46
**28**	1	93.8	4.41
**28**	10	85.16	2.56
**30a**	1	104.5	2.91

**30a**	10	94.86	4.32
**30b**	1	96.21	3.31
**30b**	10	100.6	3.31
**31**	1	96.01	3.95
**31**	10	103.1	2.8
**Maprotiline**	1	102.0	3.42
**Maprotiline**	10	65.11	4.41
**Taxol**	1	90.0	
**Taxol**	10	40.0	

[a] Cell proliferation of MUTU-1 and DG-75 cells was determined with an alamarBlue assay (seeding density 1–5 → 10^4 cells/mL per well for 96-well plates). Compound concentrations of either 1 μM or 10 μM for 24 h (MUTU-1) or 48 h (DG-75) were used to treat the cells (in triplicate) with control wells containing vehicle ethanol (1% *v*/*v*). The mean value for three independent experiments performed in triplicate is shown.

Table S5: Preliminary cell viability data for compound series **26, 27, 29, 32** and **33** in MUTU-1 Burkitt lymphoma cell line (48 h)[a]

Compound	Concentration (μM)	% Viable Cells Remaining	Error
**19a**	1	82.49	2.95
**19a**	10	5.91	1.12
**26**	1	106.2	1.85
**26**	10	94.86	4.32
**27**	1	101.7	3.62
**27**	10	93.82	4.12
**29a**	1	103.0	4.64
**29a**	10	99.01	6.87
**29b**	1	107.9	2.0
**29b**	10	106.2	3.45
**29c**	1	87.52	10.2
**29c**	10	89.22	10.65
**29d**	1	96.53	5.07
**29d**	10	102.9	1.74
**32a**	1	97.5	2.54
**32a**	10	95.54	2.73
**32b**	1	105.2	3.53
**32b**	10	82.49	2.95
**32c**	1	99.16	2.62
**32c**	10	92.65	4.85
**32d**	1	96.73	5.08
**32d**	10	95.91	4.25
**32e**	1	102.9	2.24
**32e**	10	94.86	4.32
**33a**	1	104.8	6.99
**33a**	10	103.1	6.48
**33b**	1	91.41	4.22
**33b**	10	90.13	9.23
**33c**	1	86.75	4.54
**33c**	10	77.37	4.18
**33d**	1	91.62	4.32
**33d**	10	95.58	3.42
**33e**	1	86.87	6.87
**33e**	10	85.2	9.14
**33f**	1	83.07	7.53
**33f**	10	84.75	6.42
**Maprotiline**	1	93.13	2.87
**Maprotiline**	10	72.96	3.04
**Taxol**	1	32.0	
**Taxol**	10	7.0	

[a] Cell proliferation of MUTU-1 and DG-75 cells was determined with an alamarBlue assay (seeding density $1–5 \times 10^4$ cells/mL per well for 96-well plates). Compound concentrations of either 1 μM or 10 μM for 24 h (MUTU-1) or 48 h (DG-75) were used to treat the cells (in triplicate) with control wells containing vehicle ethanol (1% *v*/*v*). The mean value for three independent experiments performed in triplicate is shown.

Table S6: Preliminary cell viability data for selected compound series **26, 27, 29, 32** and **33** in DG-75 Burkitt lymphoma cell line (48 h)

Compound	Concentration (μM)	% Viable Cells Remaining	Error
**19a**	1	94.63	3.78
**19a**	10	14.04	1.99
**26**	1	101.6	5.13
**26**	10	93.03	5.17
**27**	1	79.05	5.88
**27**	10	85.55	5.87
**29a**	1	83.61	4.37
**29a**	10	89.35	4.38
**29b**	1	93.23	3.26
**29b**	10	97.22	3.85
**29c**	1	97.34	6.39
**29c**	10	101.5	1.55
**29d**	1	86.7	2.8
**29d**	10	93.05	6.72
**32a**	1	104.2	2.82
**32a**	10	102.3	2.2
**32b**	1	94.85	2.6
**32b**	10	100.4	3.68
**32c**	1	92.52	2.03
**32c**	10	101.1	4.18
**32d**	1	91.09	7.56
**32d**	10	106.2	3.05
**32e**	1	101.6	5.13
**32e**	10	93.03	5.17
**33a**	1	90.08	5.19
**33a**	10	90.55	6.57
**33b**	1	96.22	6.24
**33b**	10	74.95	6.03
**33c**	1	69.6	12.48
**33c**	10	71.64	5.88
**33d**	1	69.44	4.03
**33d**	10	67.12	8.94
**33e**	1	86.1	7.99
**33e**	10	80.19	5.05
**33f**	1	72.53	13.04
**33f**	10	78.84	10.31
**Maprotiline**	1	102.0	3.42
**Maprotiline**	10	65.11	4.41
**Taxol**	1	90.0	
**Taxol**	10	40.0	

[a] Cell proliferation of MUTU-1 and DG-75 cells was determined with an alamarBlue assay (seeding density 1–5 → 10^4 cells/mL per well for 96-well plates). Compound concentrations of either 1 μM or 10 μM for 24 h (MUTU-1) or 48 h (DG-75) were used to treat the cells (in triplicate) with control wells containing vehicle ethanol (1% *v*/*v*). The mean value for three independent experiments performed in triplicate is shown.

Table S7: *in vitro* anti-proliferative activity of nitrostyrene compounds 11g-11k in MUTU-1 and DG-75 cell lines[a,b]

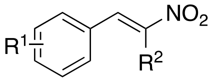

11g R^1=2-Cl, R^2=CH_3
11h R^1=3-Cl, R^2=CH_3
11i R^1=4-Cl, R^2=CH_3
11j R^1=2-Cl, R^2=CH_2CH_3
11k R^1=3-Cl, R^2=CH_2CH_3
11l R^1=4-Cl, R^2=CH_2CH_3

**Compound**	**MUTU-1 IC_{50} (μM)[b]**	**DG-75 IC_{50} (μM)[b]**	**logP**
**11g**	0.82	2.75	3.241
**11h**	0.88	2.25	3.241
**11i**	1.30	2.75	3.241
**11j**	2.18	2.05	3.77
**11k**	1.22	2.36	3.77
**11l**	1.18	3.11	3.77
**Taxol**	0.30	1.32	3.54[c]

[a] See reference [1] for our reported IC_{50} data [b]Cell proliferation of MUTU-1 and DG-75 cells was determined with an MTT assay (seeding density 1–5 × 10^4 cells/mL per well for 96-well plates). IC_{50} values are half maximal inhibitory concentrations required to block the growth stimulation of MUTU-1 and DG-75 cells. Values represent the mean ± SEM (error values × 10^{-6}) for at least three experiments performed in triplicate at 24 h. Treatment at eight different concentrations [0.001-50 μM] was used for the determination of the IC_{50} values for each compound with control wells containing vehicle ethanol (1% *v*/*v*). The mean value for three experiments is shown. [c]ChemAxon

Table S8: *in vitro* anti-proliferative activity of nitrostyrene compound **11h** in peripheral blood mononuclear cells (PBMCs), MUTU-1 and DG-75 cells

Compound	Concentration (μM)	PBMCs % viability	BL MUTU % viability	BL DG75 % viability
**11h**	10	34.08±9.587	10.26	0.038
**11h**	1	73.66±3.725	39.8	84.5

Table S9: Overlay of all nitrostyrene and nitrovinylanthracene panel compounds on maprotiline with their overlay scores and BL (MUTI-1) viability[a, b]

Compound Number	Overlay of compounds (green) with Maprotiline (pink)	Overlay (F) score	Overlay (S) score	IC_{50} (μM) MUTU-1	% viability MUTU-1 (10 μM)
**11k**	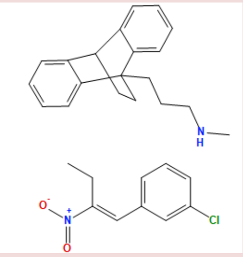 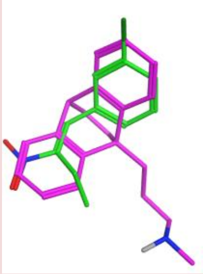	-103.1	-41.8	1.22	2.39
**11c**	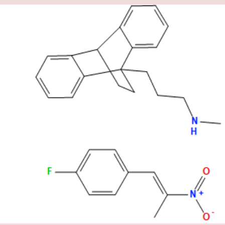 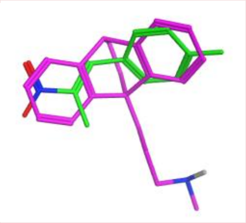	-102.8	-39.3	[c]nd	19.06
**11j**	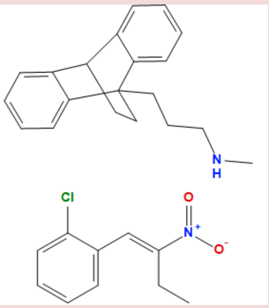 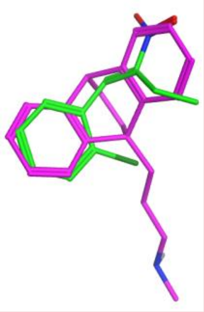	-107.2	-41.5	2.18	10.3
**19m**	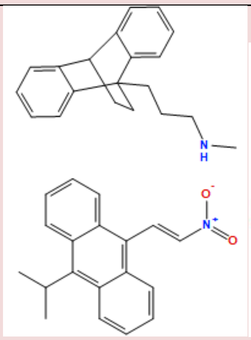 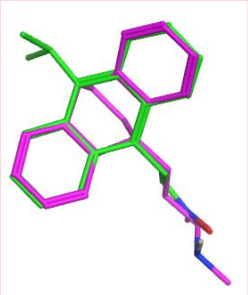	-140.4	-47.3	[c]nd	0.21

**19l**	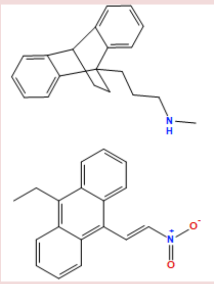 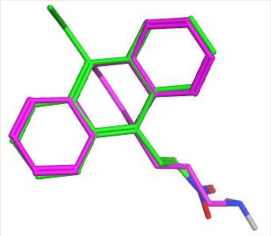	-140.2	-52.0	[c]nd	0.50
**19g**	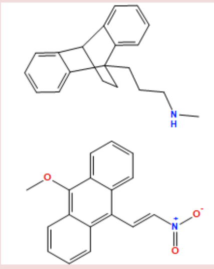 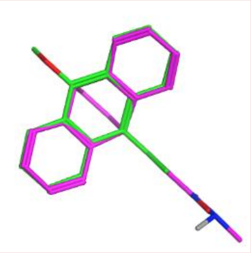	-133.8	-43.0	[c]nd	1.85
**19i**	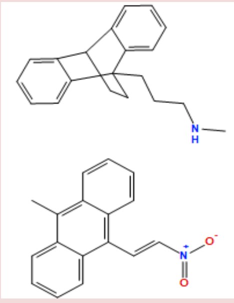 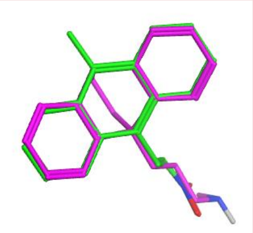	-140.4	-52.4	[c]nd	1.95
**19a**	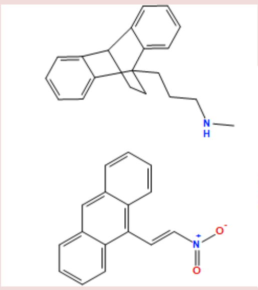 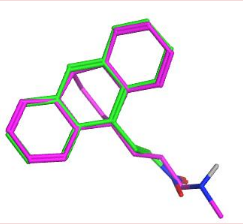	-137.5	-55.7	2.57	5.91
**11g**	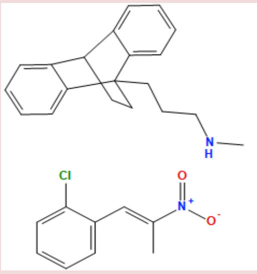 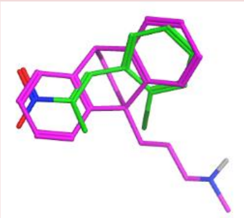	104.5	-38.2	0.82	14.91

**11h**	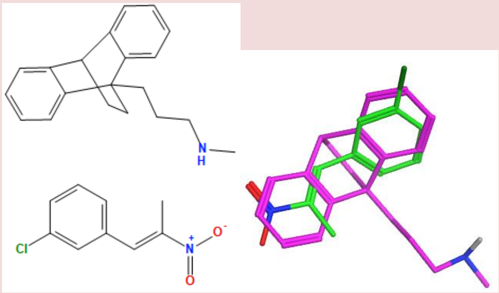	-101.1	-38.6	0.88	10.28
**11l**	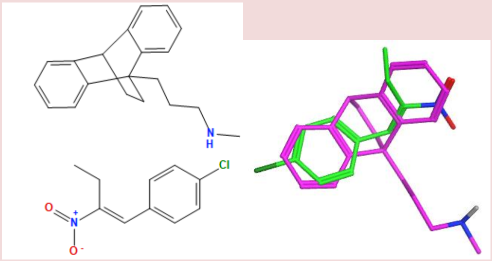	103.5	-40.4	1.18	2.61
**11i**	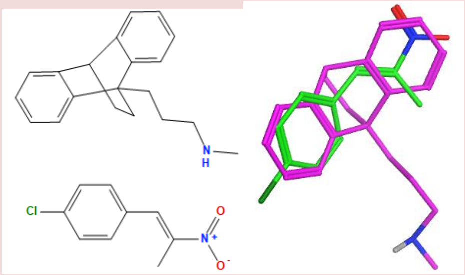	-101.1	-37.1	1.30	10.53
**19b**	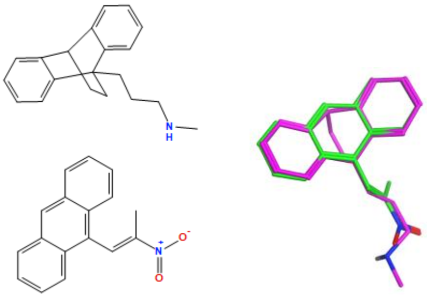	-139.2	-84.1	[c]nd	88.2
**19c**	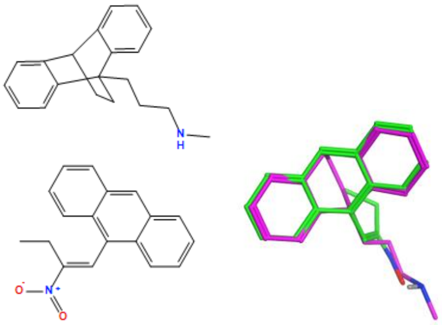	-141.4	-84.7	[c]nd	78.04

**19d**	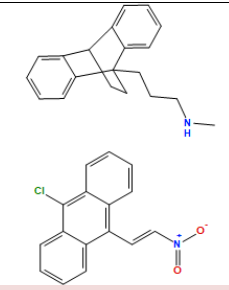 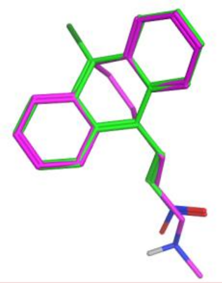	-139.9	-85.1	[c]nd	7.61
**19e**	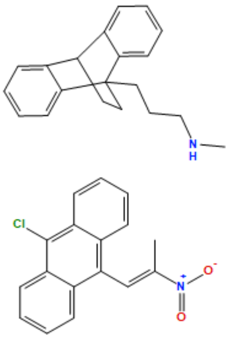 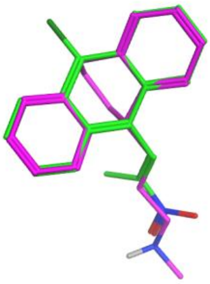	-141.2	-82.2	[c]nd	98.6
**19f**	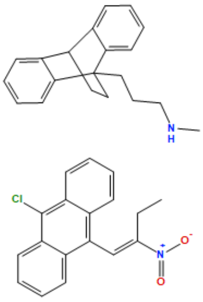 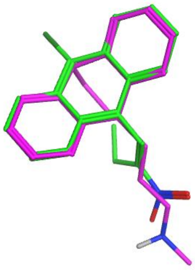	-143.3	-82.7	[c]nd	97.5
**19h**	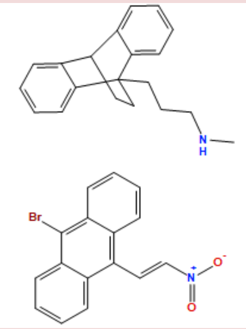 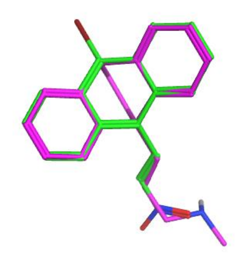	-139.5	-83.8	[c]nd	3.36
**19j**	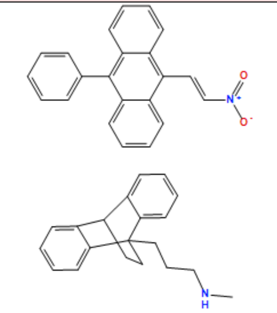 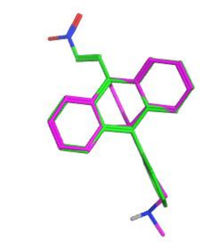	-143.8	-84.8	[c]nd	1.29

**19k**	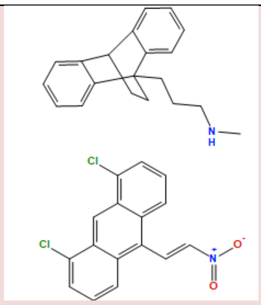 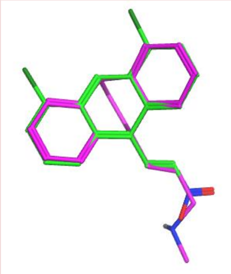	-140.1	-86.4	[c]nd	-0.18
**20a**	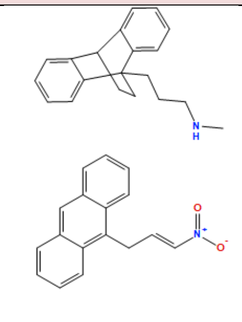 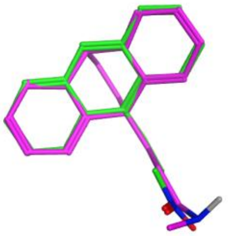	-139.5	-86.1	[c]nd	40.37
**20b**	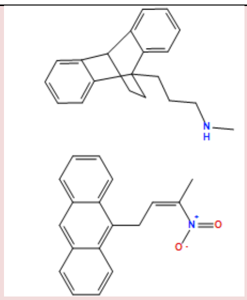 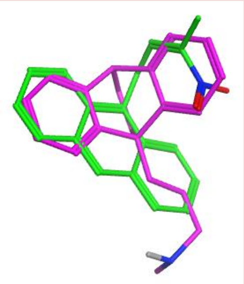	-139.4	-83.5	[c]nd	14.55
**21**	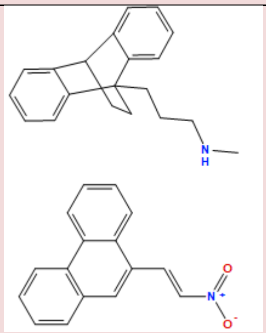 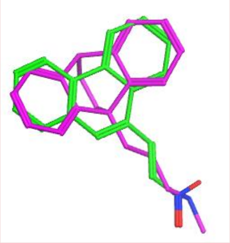	-131.1	-80.7	[c]nd	1.68
**22**	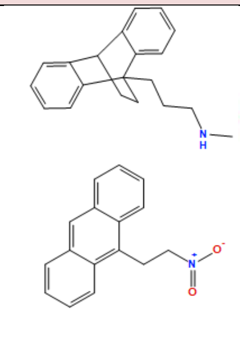 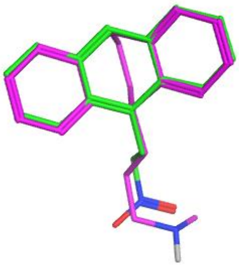	-137.5	-61.9	[c]nd	100.6

**23a**	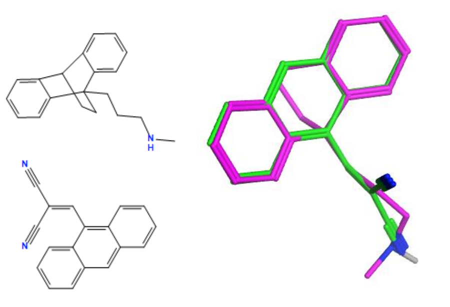	-145.2	-66.1	[c]nd	65.29
**23b**	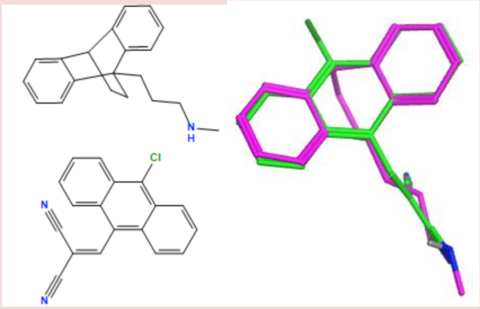	-144.6	-59	[c]nd	16.78
**23c**	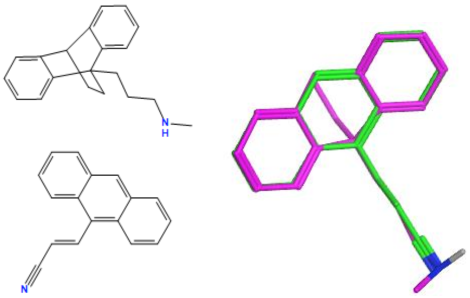	-143.8	-63.5	[c]nd	86.91
**24**	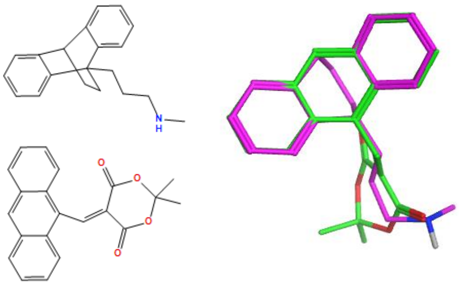	-149.5	-74.6	[c]nd	101.4
**25**	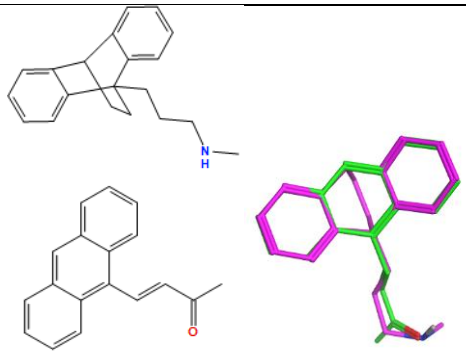	-147	-64	[c]nd	103.2

**26**	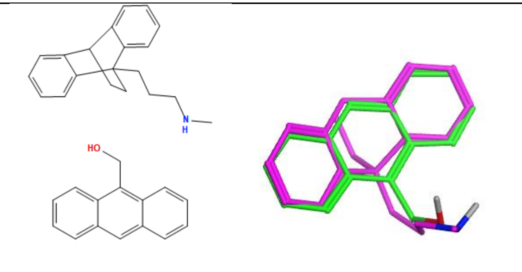	-136.4	-58.8	[c]nd	94.86
**27**	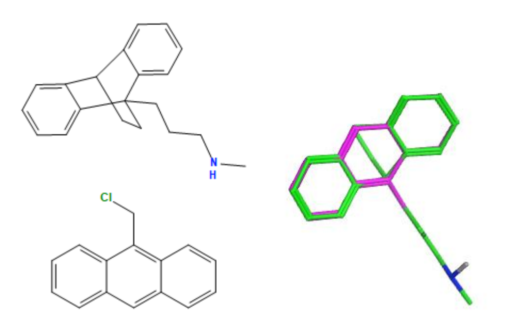	-129.9	-54.4	[c]nd	93.8
**28**	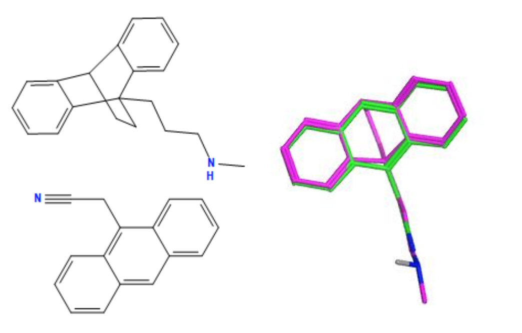	-134.9	-57.3	[c]nd	88.8
**29a**	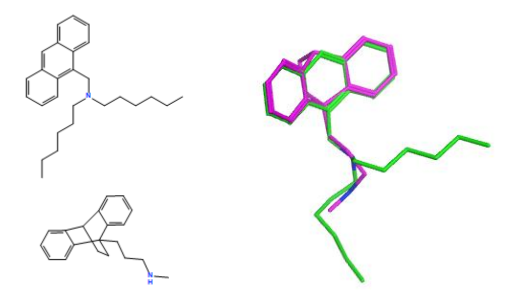	-132.8	-52.8	[c]nd	99.01
**29b**	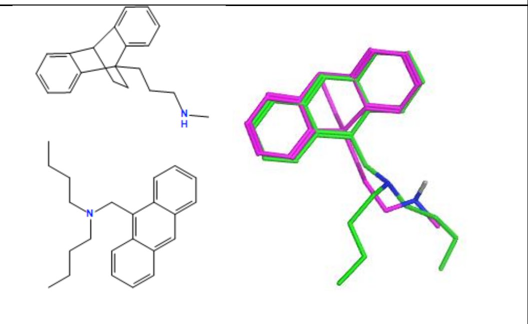	-135.9	-55.4	[c]nd	106.2

**29c**	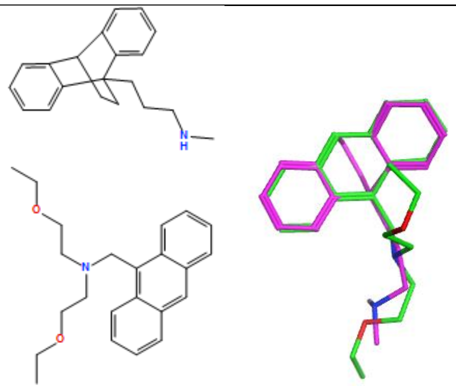	-143.8	-45.1	[c]nd	89.2
**29d**	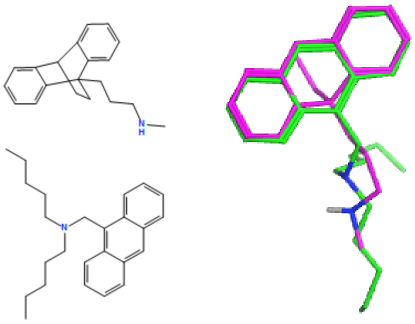	-133.8	-53.5	[c]nd	102.9
**30a**	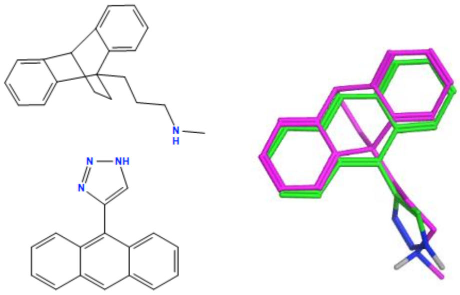	-146.9	-65.8	[c]nd	94.5
**30b**	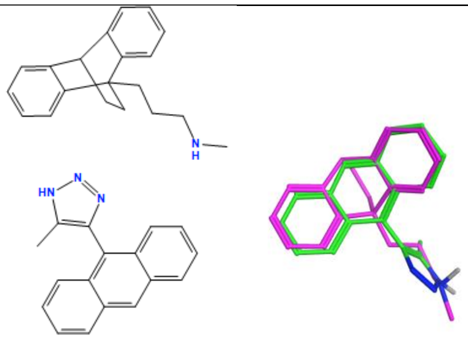	-148.1	-66.3	[c]nd	107.3
**31**	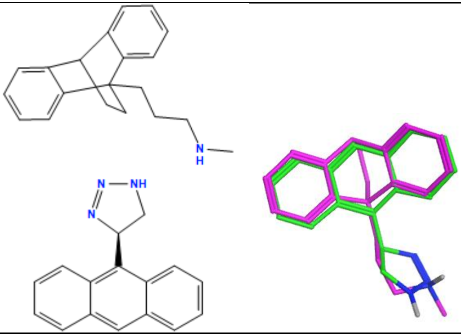	-142	-57.3	[c]nd	103.1

**32a**	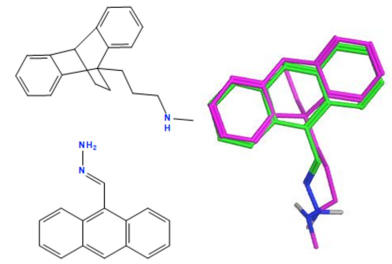	-133.4	-44	[c]nd	95.54
**32b**	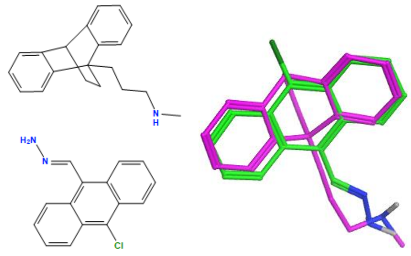	-135.8	-40.9	[c]nd	82.49
**32c**	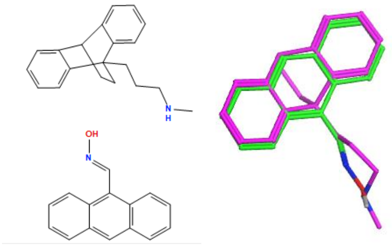	-141.9	-56.9	[c]nd	92.65
**32d**	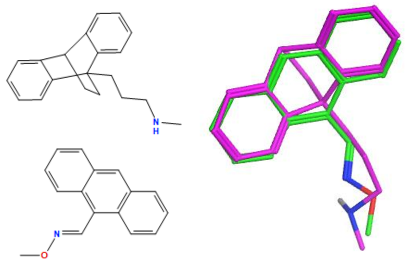	-131.5	-48.3	[c]nd	95.91
**32e**	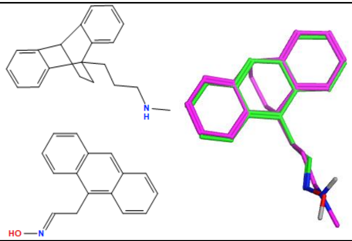	-152.5	-70.7	[c]nd	94.86
**33a**	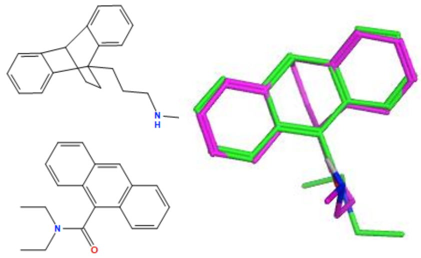	-139.9	-59.9	[c]nd	103.1

**33b**	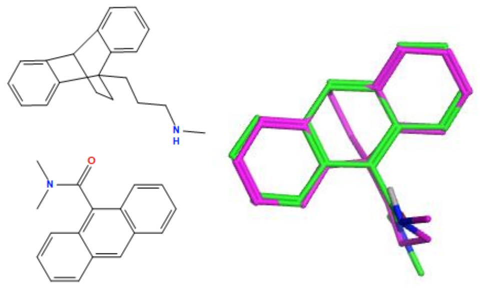	-138.5	-56.9	[c]nd	90.1
**33c**	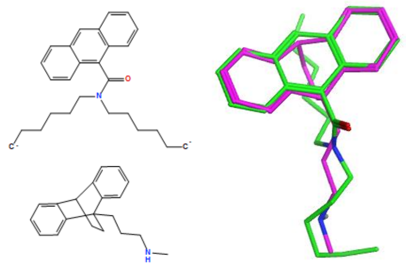	-143	-63.9	[c]nd	77.4
**33d**	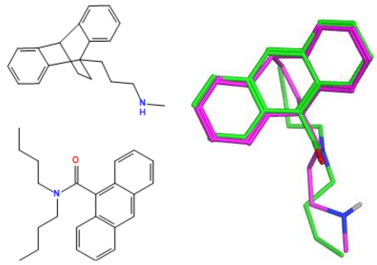	-140.5	-62.9	[c]nd	95.6
**33e**	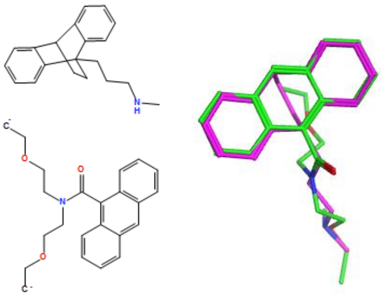	-152.9	-75.2	[c]nd	85.2
**33f**	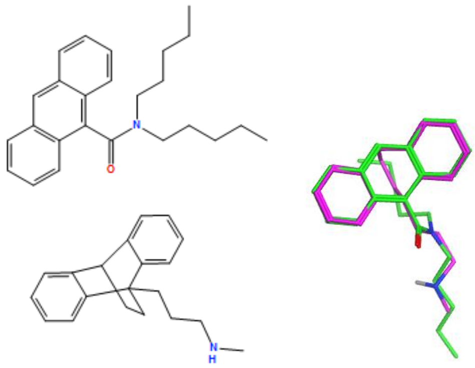	-142.1	-64.3	[c]nd	84.8

[a]Overlay of nitrostyrene and nitrovinylanthracene compound series on maprotiline with their overlay scores (F and S) in MOE 2022 and 2D images. The F column contains the similarity score (the lower the better) of the alignment. The S column has the sum of the U (not shown but is the average internal energy of the ligand) and F values. [b]The highlighted (orange) sections represent the compounds having the best IC_{50} or percentage viability values (<20% viable cells at 10 μM concentration in alamarBlue assay) in the MUTU-1 BL cell line. [c]nd Not determined

Table S10: Overlay of selected nitrostyrene and nitrovinylanthracene panel compounds on maprotiline with their overlay scores and CLL (HG-3, PGA-1) viability[a]

**Compound Number**	Overlay of compounds (green) with Maprotiline (pink)	Overlay (F) score	Overlay (S) score	IC_{50} (μM) (HG-3 cell line)	% viability (10 μM) PGA1 or HG-3 cell line
**11k**	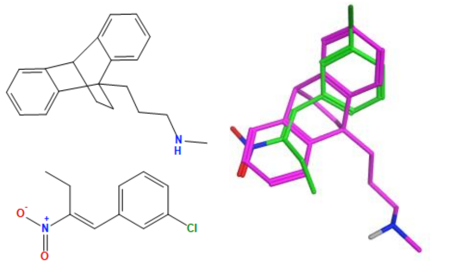	-103.1	-41.8	[b]nd	0.15
**11j**	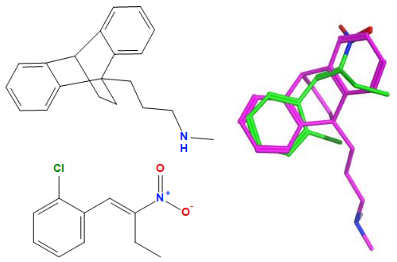	-107.2	-41.5	[b]nd	0.43
**11d**	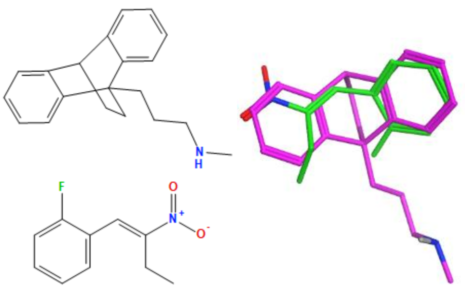	-108.9	-44.7	[b]nd	0.24
**19a**	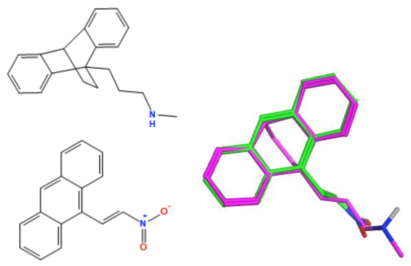	-137.5	-55.7	2.4	5.1

**19m**	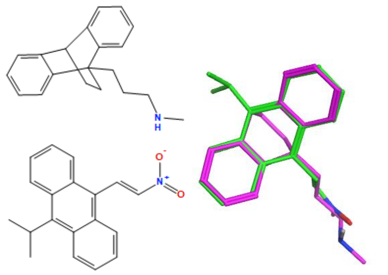	-140.4	-47.3	0.7	2.9
**19l**	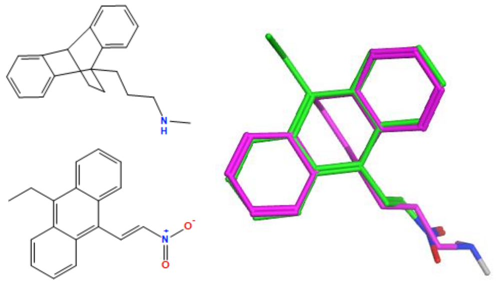	-140.2	-52.0	5.4	2.7
**19g**	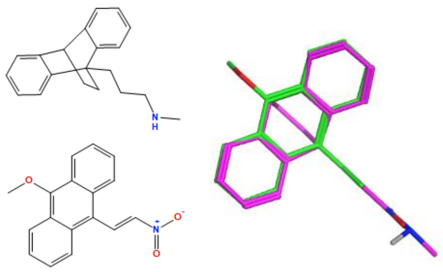	-133.8	-43.0	0.17	1.21
**19i**	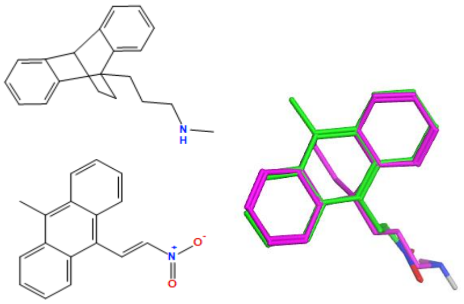	-140.4	-52.4	3.85	2.9

[a]Overlay of **11d, 11j, 11k**, **19a, 19g 19i, 19l** and **19m** on maprotiline with their overlay scores (F and S) in MOE 2022, 2D images and their percentage viability values in the PGA-1 CLL cell line and IC_{50} values in the HG-3 CLL cell line. The F column contains the similarity score (the lower the better) of the alignment. The S column has the sum of the U (not shown but is the average internal energy of the ligand) and F values. [b]nd Not determined

References

1. Byrne, A.J.; Bright, S.A.; Fayne, D.; McKeown, J.P.; McCabe, T.; Twamley, B.; Williams, C.; Meegan, M.J. Synthesis, antiproliferative and pro-apoptotic effects of nitrostyrenes and related compounds in Burkitt's lymphoma. *Med Chem* **2018**, *14*, 181-199.
2. Itamar, W., Halpern, M. Alkylation via aromatization of ketones by phase-transfer catalysis, *Synthesis,* **1979**, *3,* 177.
3. Cox, P.J.; Sim, G.A. X-ray diffraction studies of 9-methylanthraecene and 5-methylnaphthacene, *Acta Cryst.* **1979**, *B35*, 404-410.
4. Ikeda, T.; Lee, B.; Tazuke, S.; Takenaka, A. Time-resolved observation of excitation hopping between two anthryl moieties attached to both ends of alkanes: simulation based on conformational analysis. *J. Am. Chem. Soc.* **1990**, *112*, 4650-4656.
5. Vila, C.; Giannerini, M.; Hornillos, V.; Fananas-Mastral, M.; Feringa, B.L. Palladium-catalysed direct cross-coupling of secondary alkyllithium reagents. *Chem. Sci.,* **2014**, 5, 1361.
6. Iwahara, H.; Kushida, T.; Yamaguchi, A planarized 9-phenylanthracene: a simple electron-donating building block for fluorescent materials, S. *Chem. Commun.,* **2016,** *52*, 1124.
7. Martin, V.H. *Bulletin des Societes Chimiques Belges*, **1952** , *61*, 504-511.
8. Norman, R. O. C.; Waters, W. A. Action of the phenyl radical on anthracene and meso-substituted anthracenes. *J. Chem. Soc.* **1958**, 167-70.
9. Jayachitra, K.; JobePrabakar, P. C.; Ramalingam, S. Vibrational, NMR and UV–Visible spectroscopic investigation on 10-methylanthracene 9-carbaldehyde using computational calculations. *Journal of Molecular Structure,* **2020,** *1217*, 128435.
10. Jones, S., Atherton, J.C.C. An improved procedure for the preparation of 9,10-dibromoanthracene. *Synthetic Communications*, **2001**, *31*, 1799-1802.

11. Kuhn, R.; Fischer, H. Cumulenes. XIII. Amalgam-hydrogenation of cumulenes and prototropic rearrangements. *Chem. Ber.* **1961,** *94*, 3060-71.

12. House, H. O.; Ghali, N. I.; Haack, J. L.; VanDerveer, D. Reactions of the 1,8-diphenylanthracene system. *J. Org. Chem.* **1980,** *45*, 1807-17.

13. Stogryn, E. L. Synthesis and antimalarial activity of anthracene amino alcohols. *J. Med. Chem.* **1974,** 17, 563-5.

14. Becker, H. D.; Hansen, L.; Andersson, K. Synthesis and photochemical isomerization of 1,2-di-9-anthrylethanol and 1,2-di-9-anthrylethanone. *J. Org. Chem.* **1986,** 51, 2956-61.

15. Koremura, M.; Oku, H.; Shono, T.; Nakanishi, T. Relation between chemical structure and antimicrobial and insecticidal activities in organonitro compounds. VIII. Synthesis of heterocyclic nitroalkene derivatives and their activities. *Takamine Kenkyusho Nenpo* **1961,** 13, 216-21.

16. Becker, H. D.; Skelton, B. W.; Sorensen, H.; White, A. H. Photochemical *cis-trans* Isomerization of 2-Substituted 9-(2-nitroethenyl)anthracenes. X-ray structure analyses of (*E*)- and (*Z*)-9-(2-nitro-2-phenylethenyl)anthracene. *Australian Journal of Chemistry* **1989,** *42*, 593-601.

17. Nakajima, S.; Osuka, A. Synthesis of a tetrakis(9-anthryl) substituted porphyrin and intramolecular charge-transfer emission in its dication. *Tetrahedron Lett.* **1995,** *36*, 8457-60.

18. Misra, R.; Sharma, R.; Bhattacharyya, S. P. Exploring NLO response of 9,10-donor-acceptor substituted bichromophoric anthracene derivatives. *J. Comput. Methods Sci. Eng.* **2010,** *10*, 149-164.

19. Zee-Cheng, K-Y.; Cheng, C.C. Experimental tumor inhibitors. Antitumor activity of esters of ω-aryl-y-nitro-y-alken-1-ol and related compounds. *J. Med. Chem.* **1969**, *12*, 157-61

20. Daniele, M. A.; Bandera, Y. P.; Foulger, S. H. Manipulation of Forster energy transfer of coupled fluorophores through biotransformation by *Pseudomonas resinovorans* CA10. *Photochem. Photobiol.* **2012,** *88*, 129-134.

21. Lock, G.; Stach, K. Catalytic decomposition of hydrazones. I. Aromatic aldehyde hydrazones. *Ber. Dtsch. Chem. Ges. B* **1943,** *76B*, 1252-6.

22. Zhang, X. M.; Bordwell, F. G.; Bares, J. E.; Cheng, J. P.; Petrie, B. C. Homolytic bond dissociation energies of the acidic carbon-hydrogen bonds in alpha-substituted and 10-substituted 9-methylanthracenes and their related radical anions. *J. Org. Chem.* **1993,** *58*, 3051-3059.

23. Rescifina, A.; Chiacchio, M. A.; Corsaro, A.; De Clercq, E.; Iannazzo, D.; Mastino, A.; Piperno, A.; Romeo, G.; Romeo, R.; Valveri, V. Synthesis and biological activity of isoxazolidinyl polycyclic aromatic hydrocarbons: potential DNA intercalators. *J. Med. Chem.* **2006,** *49*, 709-715.

24. Kabalka, G. W.; Goudgaon, N. M. A facile aldoxime preparation via the reduction of α,β-unsaturated nitroalkenes using tin(II) chloride. *Synth. Commun.* **1988,** *18*, 693-7.

25. Jiang, L.; Fu, Y.; Li, H.; Hu, W. Single-crystalline, size, and orientation controllable nanowires and ultralong microwires of organic semiconductor with strong photoswitching property. *Journal of the American Chemical Society* **2008,** *130*, 3937-3941.

26. Hann, R. A. Use of dimethylformamide as a solvent for the Knoevenagel reaction. *Journal of the Chemical Society, Perkin Transactions 1* **1974**, 1379-1380.

27. Kumar, N. N. B.; Chakravarty, M.; Kumar, N. S.; Sajna, K. V.; Swamy, K. C. K. Allenylphosphonates with a 1,3,2-dioxaphosphorinane ring: synthesis, structures, stability and utility. *J. Chem. Sci. (Bangalore, India)* **2009,** *121*, 23-36.

28. Andrews, S. P.; Ladlow, M. Convenient preparation and use of a new analytical construct for the analysis and development of solid-phase chemistries. *J. Org. Chem.* **2003,** *68*, 5525-5533.

29. Metayer, M. Hydrogenation of 9-anthraldehyde. *Bull. Soc. Chim. Fr.* **1954**, 614-15.

30. Zhang, J-Q., Liu, J., Hu, D., Song, J., Zhu, G., Ren, H. Rapid and simple access to α-(hetero)arylacetonitriles from gem- difluoroalkenes. *Org. Lett.* **2022**, *24*, 786−790

31. Mathew, R.; Mallia, R.R.; Haridas, S.; Jacob, J.P. Effect of substituents on the fluorescence quenching of a few (anthracen-9-yl)methanamines *Journal of Photochemistry & Photobiology A: Chemistry* **2020,** *397,* 112552

32. Clayden, J.; Frampton, C. S.; McCarthy, C.; Westlund, N. Perilithiation and the synthesis of 8-substituted-1-naphthamides. *Tetrahedron* **1999,** *55*, 14161-14184.

33. Karthikeyan, J.; Yoshikai, N. Rhodium(III)-catalyzed directed peri-C-H alkenylation of anthracene derivatives. *Org. Lett.* **2014,** *16,* 4224-4227.

34. Byrne, A. J.; Bright, S. A.; McKeown, J. P.; O'Brien, J. E.; Twamley, B.; Fayne, D.; Williams, D.C.; Meegan, M.J. Design, synthesis and biochemical evaluation of novel ethanoanthracenes and related compounds to target Burkitt's lymphoma. *Pharmaceuticals* (Basel), **2020**, 13(1).

35. Lo, K.; Cornell, H.; Nicoletti, G.; Jackson, N.; Hügel, H., [35]. *Appl. Sci*., **2012**, *2*, 114-128.

36. Si, T.; Kim, H.Y.; Oh, K., Substrate promiscuity of ortho-naphthoquinone catalyst: catalytic aerobic amine oxidation protocols to deaminative cross-coupling and N-nitrosation. *ACS Catalysis*, **2019**, *9*, 9216-9221.

37. Sen, P. K.; Sikha; L.; Chattopadhyay, G.; Mandal, D.K.; Oxidation of 10-methoxy-9-anthraldehyde with various oxidizing agents in protic and aprotic media, *Indian Journal of Chemistry* **1989**, *28B*, 978-9.
